# A Molecular View of Kinetochore Assembly and Function

**DOI:** 10.3390/biology6010005

**Published:** 2017-01-24

**Authors:** Andrea Musacchio, Arshad Desai

**Affiliations:** 1 Department of Mechanistic Cell Biology, Max Planck Institute of Molecular Physiology, Otto-Hahn Straße 11, Dortmund 44227, Germany; 2 Centre for Medical Biotechnology, Faculty of Biology, University Duisburg-Essen, Essen 45117, Germany; 3 Ludwig Institute for Cancer Research, La Jolla, CA 92093, USA; 4 Department of Cellular & Molecular Medicine, 9500 Gilman Dr., La Jolla, CA 92093, USA

**Keywords:** centromere, kinetochore, cell division, mitosis, meiosis, KMN, CCAN, CENP-A

## Abstract

Kinetochores are large protein assemblies that connect chromosomes to microtubules of the mitotic and meiotic spindles in order to distribute the replicated genome from a mother cell to its daughters. Kinetochores also control feedback mechanisms responsible for the correction of incorrect microtubule attachments, and for the coordination of chromosome attachment with cell cycle progression. Finally, kinetochores contribute to their own preservation, across generations, at the specific chromosomal loci devoted to host them, the centromeres. They achieve this in most species by exploiting an epigenetic, DNA-sequence-independent mechanism; notable exceptions are budding yeasts where a specific sequence is associated with centromere function. In the last 15 years, extensive progress in the elucidation of the composition of the kinetochore and the identification of various physical and functional modules within its substructure has led to a much deeper molecular understanding of kinetochore organization and the origins of its functional output. Here, we provide a broad summary of this progress, focusing primarily on kinetochores of humans and budding yeast, while highlighting work from other models, and present important unresolved questions for future studies.

## 1. An Overview of Kinetochore Structure and Functions

**Figure 1:**
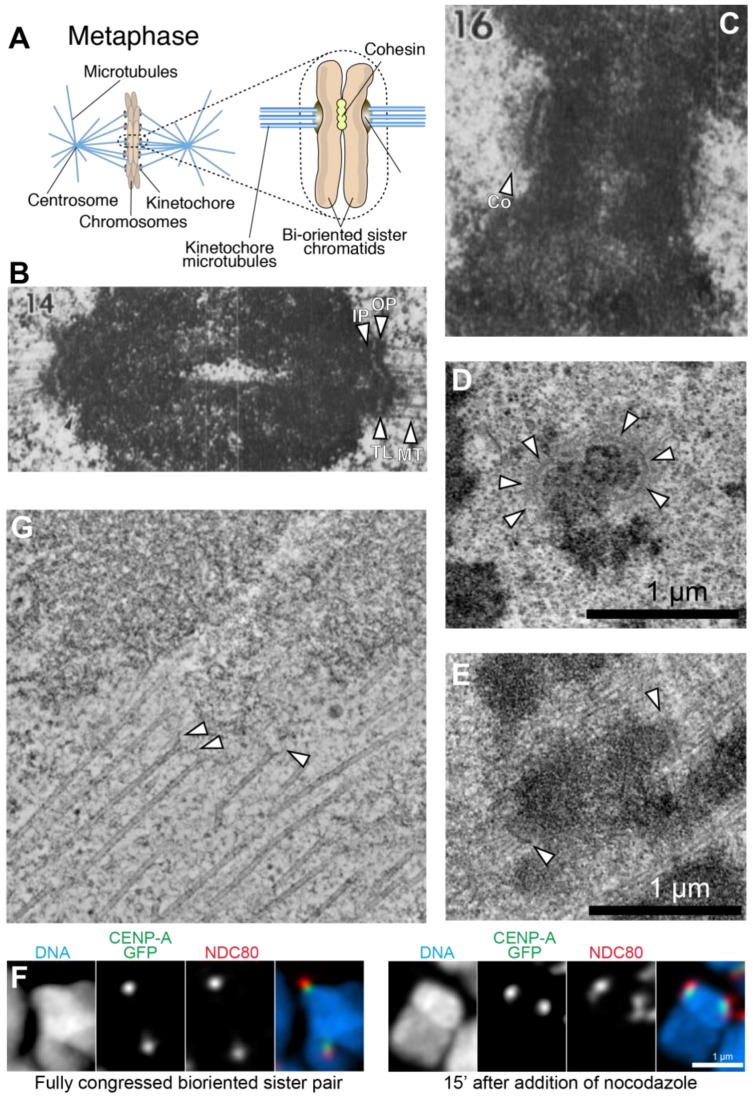
Kinetochore morphology in vertebrate cells (**A**) Schematic showing the attachment of chromosomes to spindle microtubules through kinetochores; (**B**) Early work on the kinetochore identified inner and outer plates, separated by a translucent layer. Microtubules terminate end-on on the kinetochore outer plate. Arrowheads indicate inner plate (IP), outer plate (OP), translucent layer (TL), and kinetochore microtubules (MT). Image reproduced with permission from reference [7]; (**C**) The corona (Co) is a fibrous structure that is more clearly visible on kinetochores prior to microtubule attachment. Image reproduced with permission from reference [7]; (**D**,**E**) Prior to microtubule attachment (**D**), vertebrate kinetochores adopt a crescent-like shape. The latter is not visible on fully congressed and bi-oriented kinetochores. Images courtesy of Alexey Khodjakov. See also reference [8]; (**F**) Left: at metaphase, the distributions of two proteins in the inner and outer kinetochores (NDC80 and CENP-A respectively), are similar; Right: After treatment with a microtubule-depolymerizing drug (nocodazole), proteins in the corona (not shown) and in the outer kinetochore undergo an expansion and form the crescent-like shape already shown in **D**. Image courtesy of Alexey Khodjakov. See also reference [8]; (**G**) A prometaphase PtK2 cell prepared for electron microscopy by high-pressure freezing and freeze-substitution in glutaraldehyde and Osmium tetroxide. The cell was then embedded in plastic, serial-sectioned with 300 nm sections, and imaged by serial tilting. A 3D reconstruction was computed by back-projection, using the IMOD software package. The slice shown here is about 5 nm thick, and represents the average of two consecutive tomographic planes. Arrowheads indicate slender fibrils connecting the end of microtubules to the kinetochore. Image courtesy of J. Richard McIntosh.

In eukaryotes, the kinetochore is a proteinaceous multi-subunit assembly whose main function is to generate load-bearing attachments of sister chromatids (the replicated chromosomes held together by the protein complex cohesin) to spindle microtubules during cell division (mitosis or meiosis) (Figure 1A). Kinetochores couple sister chromatids to dynamic microtubules during congression and anaphase, allowing their separation and partition to the daughter cells [1,2,3].

Kinetochores assemble on a specialized chromatin locus named the centromere (which, when large enough to be observed, coincides with the primary constriction on chromosome spreads in karyotype analysis) [4,5,6]. Even if the name ‘centromere’ implies a position at the center of the chromosome, centromeres in different organisms can occupy very different positions, and are generally defined as metacentric (if they are in the middle of the chromosome), acrocentric (if they separate chromosome arms of different length), or telocentric (if they are positioned very close to a chromosome’s end). In organisms, such as nematodes, several insects, and lower plants, centromeres extend along the entire length of the chromosome (so-called holocentric centromeres, in opposition to spatially delimited monocentric centromeres). The size of the chromosome segment required to assemble a functional kinetochore varies wildly from species to species, from ~125 base pairs (bps) in *Saccharomyces cerevisiae* to one or more million bps in humans. Most centromeres are defined by a specific chromatin signature rather than a specific DNA sequence (with notable exceptions discussed in Section 2). This property is generally referred to as epigenetic specification of centromeres [4,5,6]. Despite the considerable compositional and positional variety of centromeres, a common molecular architecture is clearly discernible in kinetochores across the eukaryotic world, with the significant known exception of kinetoplastids (see Section 8). 

Besides mediating interactions with spindle microtubules, kinetochores are mechanosensors that control stability of microtubule attachment to favor the bi-orientation of sister chromatids (or of the bivalents during meiosis), instead of incomplete or incorrect configurations such as mono-orientation, syntelic attachment, or merotelic attachment [9]. This property of kinetochores is generally referred to as error correction, and its molecular basis remains rather poorly understood. The pioneering experiments of Nicklas and colleagues [10], as well as more recent functional analyses [11,12,13], suggest that the development of tension within the kinetochore or between sister kinetochores contributes to discerning correct attachment from incorrect ones (see chapter by Lampson and Grishchuk, reference [14]). Kinetochores also regulate the spindle assembly checkpoint (SAC, also named the metaphase checkpoint), a feedback mechanism required to couple the initiation of mitotic exit with the completion of sister chromatid bi-orientation [15,16]. The trigger of mitotic exit is the inactivation of mitotic Cyclin-dependent kinase (CDK) activity and the activation of the protease activity that eliminates sister chromatid cohesion. Both processes are regulated by Ubiquitin-dependent proteolysis, and the SAC inhibits this regulated proteolysis to prevent premature mitotic exit in presence of unattached or improperly attached kinetochores (see chapter by Ajit Joglekar, reference [17]). There is overlap between the functions of the SAC as a mechanism to gain time when chromosome attachment is incomplete or erroneous, and the function of the error correction apparatus that aims to favor bi-orientation. Indeed, common molecular machinery regulates these processes, at least at the apex of the pathway.

The ultrastructure of the vertebrate kinetochore is described based on early electron microscopy (EM) studies employing glutaraldehyde fixation that identified kinetochores as trilaminar structures, approximately 250 nm wide and 80 nm deep, with an electron-opaque inner plate juxtaposed to the centromeric chromatin, a translucent gap layer, and an electron-opaque, chromatin-distal outer plate apparently embedding the plus ends of spindle microtubules (defined as end-on attachment, Figure 1B) [7,18]. Furthermore, in the absence of microtubules, a fibrous structure named the corona becomes apparent externally to the outer plate [19,20,21,22,23] (Figure 1C). The corona, which is not morphologically discernable following microtubule binding, triggers a significant expansion of the kinetochore in a crescent-like shape [7,8,24,25,26] (Figure 1D,E). Studies with improved fixation (high pressure freezing followed by freeze substitution) failed to confirm the existence of a clearly defined trilaminar plate structure in the kinetochore, and have rather redefined the kinetochore as a disordered fibrous mesh in which the plus ends of microtubules are embedded [8,27,28]. Depolymerizing protofilaments of microtubules were shown to establish connections to the kinetochore through slender fibrils [29] (Figure 1F). 

A significant limitation in our understanding of kinetochores until the early 2000s was that a molecular description of their architecture was largely missing. The advent of mass spectrometry-based proteomics and functional genomics has led to substantial progress in the identification of kinetochore subunits and sub-complexes, their reconstitution and purification, and their structural characterization at high-resolution by X-ray crystallography and EM [3]. In particular, the structure of most of the components of the outer kinetochore is now known, or can be inferred through cross-linking experiments, and parts of the inner kinetochore are also beginning to be characterized. Below, we first present a brief summary of studies on the centromere and its epigenetic definition. We then review progress toward defining the structural organization of kinetochores, with references to older foundational work and to accompanying chapters in this issue that focus on the functional output of kinetochores, including microtubule-dependent force generation, error correction, and the SAC (see references [14,17,30]). In our discussion of kinetochore structural organization, we will focus on human kinetochores and lessons from recent biochemical reconstitution efforts but will refer to work in other models to highlight important parallels/differences, and also to present questions that emerge from cross-model comparisons.

## 2. The Centromere

**Figure 2:**
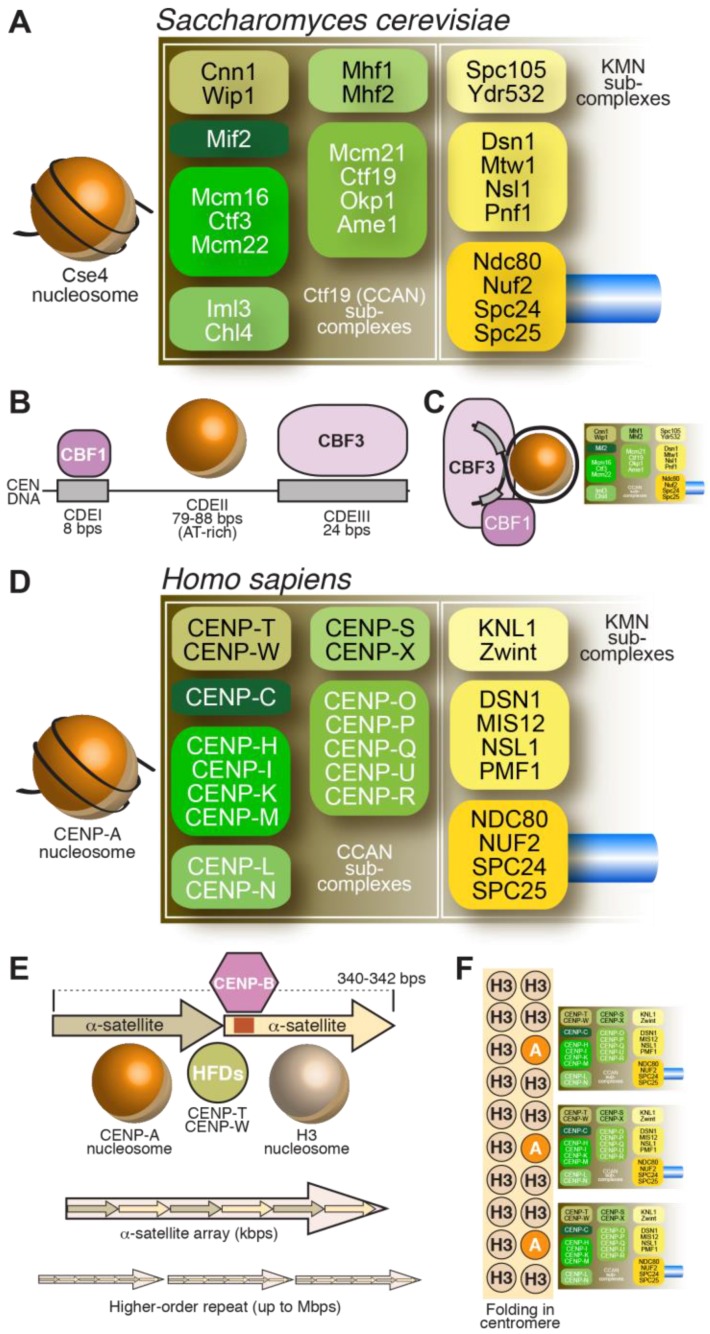
Schematic summary of the structural organization of budding yeast and human kinetochores. Related colors highlight conserved components/complexes. (**A**) Schematic of the *S. cerevisiae* kinetochore with subunit names; (**B**) The *S. cerevisia*e centromere (CEN) DNA is stereotyped and contains CDEI, CDEII, and CDEIII regions, which bind CBF1, Cse4^CENP-A^, and CBF3, respectively; (**C**) Folding of CEN DNA around a Cse4 nucleosome brings CBF1 and CBF3 in close proximity; (**D**) Schematic of the *H. sapiens* kinetochore. Orthologous complexes are shown in the same order as in (**A**); (**E**) The unit of human centromere assembly may consist of a pair of α-satellite repeats, each precisely wrapping around a nucleosome. One of the two α-satellite repeats carries a CENP-B box. The CENP-TW complex may interact in the inter-nucleosomal region through its histone-fold domain (HFDs) [77,78]. Repeats of this unit give rise to α-satellite arrays, which in turn may organize themselves in higher order repeats (HORs); (**F**) The human centromere arises from folding of centromeric chromatin in three dimensions to facilitate the participation of several CENP-A nucleosomes in kinetochore assembly.

As discussed in the previous section, the centromere is the specialized chromatin region on which kinetochores assemble. The DNA sequence at the centromere, however, varies considerably from organism to organism. The short *cis*-acting DNA segments of *S. cerevisiae* centromeres (usually designated as CENs) have overall conserved sequence features among the 16 chromosomes and are sufficient for kinetochore assembly [31,32,33,34,35]. This type of centromere, found in *S. cerevisiae* and related fungi, is referred to as a point centromere [36] (Figure 2A–C). The complexity of centromeres in most other organisms, however, vastly exceeds that of the *S. cerevisiae* centromere. In the majority of model systems studied to date, centromeres consist of highly repetitive DNA elements, including retro-transposons or tandem repeat arrays, or combinations of both [4,6,37]. These centromeres span chromosome regions in a range from tens of thousands to millions bps, and have therefore been defined as regional centromeres [36]. For instance, human centromeres consist of a large number of tandem 171-bps repeats, called α-satellite repeats, which extend within domains of ~0.2–4.0 Mbps [38] (Figure 2D–F). Tandem repetitive sequences, unrelated to those in humans, are also identified at centromeres of mice, fission yeast, flies, and plants, among others [4,6,37]. These complex regional centromeres have a central portion where the kinetochore is assembled, flanked by pericentromeric regions that are often also repetitive, heterochromatic in nature and accumulate cohesin complexes.

In contrast to *S. cerevisiae*, it has not been possible to identify, in these larger and more complex centromeres, a univocal relation between the underlying sequence of the centromere and the ability to seed a kinetochore [4,6,37]. For instance, conversion of a non-functional centromere to a functional one on mini-chromosomes can occur in the absence of apparent sequence, structural, or chemical changes in *S. pombe* [39]. Stably inherited dicentric chromosomes (chromosomes with two distinct repeat arrays normally associated with centromere function) invariably show inactivation of one of the two predicted centromeres, indicating that the DNA sequence is insufficient to establish the kinetochore [40,41]. On the same line, functional neo-centromeres can form at euchromatic regions of human supernumerary marker chromosomes in the absence of alphoid DNA [42,43,44], showing that repetitive DNA is not necessary for a functional centromere. Similarly, acentric (i.e., centromere lacking) chromosome fragments produced by irradiation can be transmitted quite faithfully in *D. melanogaster* cells because they acquire neo-centromere activity at non-repetitive sequences [45]. That the presence of repetitive sequences is not an absolute requirement for centromere identity is also supported by the observation that centromeres of several organisms are devoid of them [46,47,48,49,50]. Repetitive sequences, however, are likely to contribute to the stabilization of centromere organization and function. Evolutionarily new centromeres (ENCs), which are initially generated by centromere repositioning in non-repetitive, “gene desert” regions of the genome without additional chromosomal changes, re-acquire repetitive DNA sequences in short evolutionary times [51,52,53].

**Figure 3:**
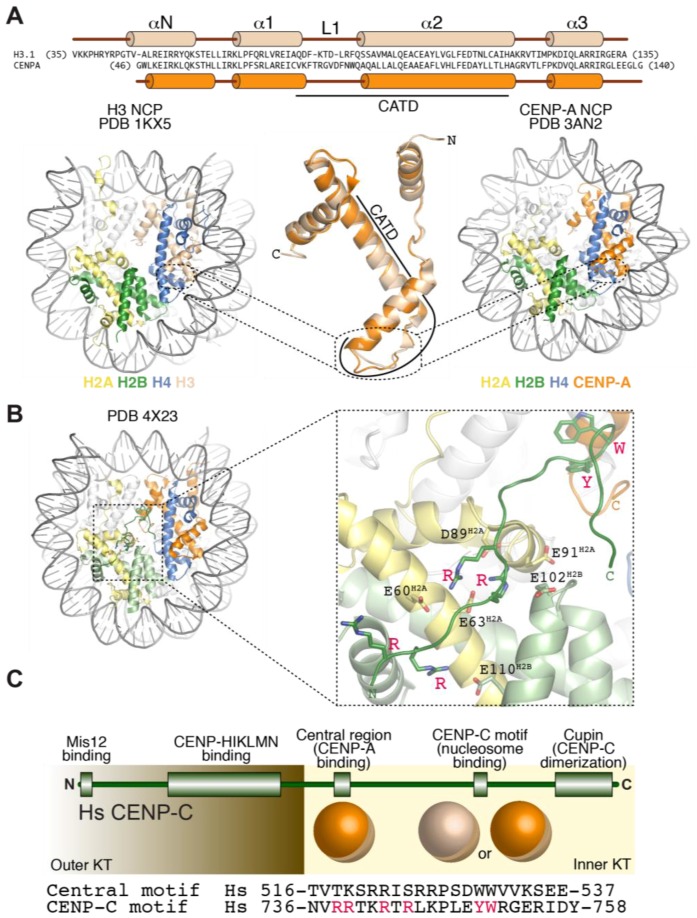
The CENP-A nucleosome and its specific recognition by CENP-C. (**A**) Comparison of H3 and CENP-A primary, secondary, tertiary, and quaternary structure. Sequence and structure changes concentrate in the N-terminal region, in the L1 segment of the CATD, and in the C-terminal region; (**B**) Structure of the complex of the CENP-C motif bound to a nucleosome containing a chimeric histone H3 with grafted hydrophobic C-terminal peptide of CENP-A [79]; (**C**) Scheme illustrating the organization of CENP-C as a “blueprint” for kinetochore assembly along the outer to inner kinetochore axis [80]. The H3 nucleosome structure is from *X. laevis*, the CENP-A nucleosome structure is human, and the CENP-C motif-bound structure has a *Drosophila* nucleosome core particle (in which the human CENP-A tail was grafted onto H3) bound to a rat CENP-C motif.

Collectively, the observations discussed in the previous paragraph provided the foundation for the idea that centromeres in most organisms are defined epigenetically rather than through specific DNA sequences [54,55,56]. Over the years, the search for crucial determinants of epigenetic specification of centromere identity has narrowed to CENP-A (also called CenH3) [57,58]. CENP-A is a histone H3 variant [59,60,61] (Figure 3A). (Specific features of CENP-A that distinguish it from canonical H3 are discussed in Section 2.) With some exceptions [62], CENP-A is present at functional centromeres, from *S. cerevisiae* (where it is called Cse4) to humans [61,63,64]. CENP-A is required for kinetochore recruitment of all other kinetochore components [65,66,67,68,69], and is sufficient to promote kinetochore assembly when targeted artificially to ectopic locations [70,71,72,73,74,75,76].

While the association of CENP-A, a crucial epigenetic factor, with the sequence-specific yeast centromere may seem counterintuitive, it is important to note that CENP-A not only serves as an epigenetic mark but also functions as the foundation for kinetochore assembly (as discussed in Section 3), and this latter function is retained by *S. cerevisiae* CENP-A^Cse4^. In *S. cerevisiae*, the CEN DNA-binding CBF3 complex helps target CENP-A^Cse4^, making propagation of CENP-A nucleosomes genetically specified and restricted to a specific location. The concept of epigenetic specification in other species relates to the fact that the presence of CENP-A on a defined segment of DNA is (largely) sequence independent, yet extremely stable and self-propagating at that particular locus (the centromere) through multiple cell generations. The molecular basis for this phenomenon is discussed in Section 7.

Why do centromeres in most species rely on an epigenetic identity and vary so significantly in sequence despite their essential role in cell division? And why do active centromeres accumulate repetitive DNA sequences? While definitive answers to these questions remain to be obtained, some current hypotheses are mentioned here in brief. With regards to the first question, the ‘centromere drive’ hypothesis posits that asymmetry of chromosome segregation in oocytes, where only a quarter of the genome is transmitted to the egg while the rest is discarded in polar bodies, leads to a genetic conflict that drives rapid centromere evolution [81]. This hypothesis has received support from evidence of adaptive evolution in centromere/kinetochore proteins [82,83] and from analysis of centromere activity in mouse strains with variation in centromeric repeats [84]. A different but not mutually exclusive idea is that the foundation for kinetochore assembly requires a chromatin state that is defined largely by architectural instead of sequence constraints, thereby reducing selection pressure to maintain specific sequences. Provided that this chromatin architecture can be inherited through division, such a model would explain centromere variation also in species that lack asymmetric segregation during meiosis. For the question regarding presence of repeats at centromeres, neocentromeres lacking repeats have been shown to be more sensitive to missegregation and to localize lower amounts of the error correction machinery [85], implicating repeats in segregation accuracy. One hypothesis, based on studies in *S. pombe* [86,87] is that repetitive sequences trigger heterochromatin formation, which in turn promotes cohesin complex enrichment that both mechanically strengthens the centromere and promotes localization of error correction machinery. Such a model would link optimal centromere functionality to repeat accumulation, potentially accounting for why repeats have independently accrued at centromeres of divergent species.

## 3. The Inner Kinetochore

### 3.1. The CCAN

Despite considerable diversity of centromere organization in different organisms, kinetochores share significant similarity in their biochemical composition in evolution [88,89]. Early inroads into the identification of proteins present at kinetochores were made when sera from patients diagnosed with the autoimmune syndrome CREST (Calcinosis, Reynaud’s syndrome, Esophaegal dysmotility, Sclerodactyly, Telangiectasia) detected centromeres in cells [90]. Subsequent work with these anti-centromere antibodies (ACA) let to the identification of three antigens, which were named CENP-A, CENP-B, and CENP-C, where CENP stands for centromeric protein [57,58]. Subsequent work led to the identification of the coding sequence of the polypeptides to which these antigens belonged [61,91,92]. In the following years, additional human CENPs were identified, including CENP-H and CENP-I, the latter related to a previously identified fission yeast protein, Mis6 [93,94,95]. Subsequent analyses of the CENP-A, CENP-H, and CENP-I associated proteomes in vertebrate cells led to the identification of several new CENPs, including CENP-K, CENP-L, CENP-M, CENP-N, CENP-O, CENP-P, CENP-Q, CENP-R, CENP-S, CENP-T, CENP-U (also known as CENP-50), CENP-W, and CENP-X [96,97,98,99,100,101]. This set of CENPs is now collectively identified as constitutive centromere associated network (CCAN), a name emphasizing localization of at least a subset of CCAN subunits at centromeres throughout the cell cycle [100,102]. The CCAN proteins localize to the most chromatin-proximal region of the kinetochore [103,104,105,106] (Figure 2D). 

Biochemical reconstitution and reciprocal dependency for kinetochore recruitment indicate that the majority of the CCAN assembly can be subdivided into 4 discrete entities (Figure 2D): the CENP-LN complex [107,108,109,110], the CENP-HIKM complex [80,110,111,112,113,114,115], the CENP-OPQRU complex [100,115,116] and the CENP-TWSX complex [100,101]. These CCAN sub-complexes are probably constitutive, i.e., the stability of their subunits depends critically on reciprocal interactions in their cognate complex. These building blocks further interact as discussed later in this section.

Most of the CCAN subunits have orthologs in *S. cerevisiae* [107,114,115,117,118,119], which are collectively identified as the Ctf19 complex (Figure 2A). A notable exception is the 4-subunit CBF3 complex (Figure 2B,C), a cognate binding partner of the CEN DNA of *S. cerevisiae* [34]. CEN DNA contains three major regions of sequence similarity, named CDEI, CDEII, and CDEIII [35]. CDEII, which has an AT content of ~90%, is the binding site for the CENP-A^Cse4^ nucleosome (discussed in more detail in Subsection 3.2) [120,121,122], whereas CDEI and CDEIII bind respectively to the general transcription factor Cbf1 and to CBF3 [34,123,124,125]. Furthermore, CBF3 and Cbf1 interact, establishing a bridge between CDEI and CDEIII that contains the CENP-A^Cse4^ nucleosome [126,127,128,129]. While the CENP-A^Cse4^ nucleosome may be intrinsically left-handed (see Section 3.2), it has been proposed that CBF3 may configure a right-handed DNA loop [130,131,132]. 

Surprisingly, CCAN subunits, with the notable exception of CENP-C, have not been found to date in certain lineages, e.g., in *D. melanogaster* or *C. elegans* and related species [133]. This apparent loss in species that rely on CENP-A-based kinetochores for chromosome segregation, together with variation in the phenotypic impact of removal of CCAN subunits in species where they are present, highlights that much still remains to be understood about the structural and functional contributions of these four CCAN complexes at the kinetochore.

### 3.2. Structural Organization of the CENP-A Nucleosome

CENP-A retains several properties of histone H3 (Figure 3A). It interacts tightly with histone H4, and is incorporated in vitro and in vivo into canonical octameric nucleosomes with histones H2A and H2B that share many structural features of the canonical H3-containing nucleosomes, including a left-handed DNA writhe [79,134,135,136,137,138,139,140,141,142,143,144]. CENP-A nucleosomes have looser terminal contacts in comparison to H3 nucleosomes and protect a shorter DNA core (~100–120 bps) in nuclease protection assays, a property enhanced by CENP-C binding [77,120,136,141,145,146,147].

Alternative models for the organization of CENP-A-containing nucleosomes have been proposed in recent years, and readers are referred to comprehensive recent discussions [148,149]. Given the importance that direct recognition of CENP-A plays in kinetochore assembly and stability, understanding the effective organization of the CENP-A nucleosome and its dynamic changes during the cell cycle is of great importance. To date, successful in vitro reconstitution of physical interaction of inner kinetochore proteins with CENP-A has been limited to octameric nucleosomes [79,110,141,150,151]. These high-affinity interactions occur at thermodynamic equilibrium and may account for the remarkable long-term stability of CENP-A in chromatin in vivo [141,152,153]. Thus, thermodynamic stability is a benchmark against which alternative models for the role of CENP-A in kinetochore assembly will have to be tested. This consideration does not detract from the possibility that structural changes in the organization of the CENP-A nucleosome occur during the cell cycle (e.g., during DNA replication) [148].

### 3.3. Recognition of CENP-A by CCAN Subunits

So far, two CCAN subunits, CENP-C and CENP-N (Figure 2D), have been found to interact directly with CENP-A and exhibits specificity for CENP-A versus H3 nucleosomes [76,79,110,140,150,151]. CENP-N binds directly to the CENP-A centromere-targeting domain (CATD, Figure 3A) of CENP-A [150]. The CATD comprises residues in the α1–α2 (L1) loop and the α2 helix of CENP-A and harbors the highest concentration of sequence differences between CENP-A and H3, with a preponderance of these in the L1 loop, which is also the only solvent-exposed region of the CATD (Figure 3A). The CATD is required for incorporation in centromeric chromatin, and is also sufficient, when grafted onto the equivalent position of H3, for loading of the H3 chimera to centromeres [135,139]. The latter property likely reflects a second requirement of the CATD (besides CENP-N binding), the interaction with a specific CENP-A chaperone required for incorporation of CENP-A into chromatin (see Section 7).

CENP-C, on the other hand, interacts with the acidic patch of H2A and H2B as well as with the divergent C-terminal tail of CENP-A [76,79,140,154] (Figure 3B). Two sequence-related regions of CENP-C, the central region and the CENP-C motif, have been implicated, each on its own right, in the interaction with the CENP-A nucleosome (Figure 3C) [79]. The central region and the CENP-C motif each encompass ~25 residues, and contain several conserved positively charged residues near their N-terminus and two aromatic residues near their C-terminus. The N-terminal positively charged region interacts with the acidic patch of H2A and H2B on the CENP-A nucleosome, a region that has been implicated in the interaction of canonical H3 nucleosomes with different target proteins [155,156,157]. The aromatic residues, on the other hand, interact with the C-terminal tail of CENP-A, which is known to be necessary for CENP-C binding [69,70,76,151,154,158] (Figure 3C). Despite relatively modest evolutionary sequence conservation, a common trait of the CENP-A C-terminal tail is that its sequence is considerably more hydrophobic than that of H3 (Leu-Glu-Glu-Gly-Leu-Gly and Glu-Arg-Ala in human CENP-A versus H3, respectively, Figure 3A). Thus, rather than a specific amino acid sequence, the higher hydrophobicity of the C-terminal tail of CENP-A may be key for specific recognition by CENP-C [79].

Despite their being related in sequence, the central region and the CENP-C motif of CENP-C do not have the same potential for kinetochore recruitment. The central region is necessary and sufficient to promote CENP-A nucleosome binding in vitro and kinetochore targeting in vivo [79,151,159,160,161,162,163,164,165,166]. The CENP-C motif, on the other hand, is insufficient for kinetochore targeting, but can be recruited to kinetochores as part of a larger C-terminal fragment capable of homo-dimerization with endogenous CENP-C through a C-terminal ‘Mif2-homology’ cupin-like domain [162,163,164,167,168]. Furthermore, while not sufficient for centromere recruitment in the absence of endogenous CENP-C, the CENP-C motif and the dimerization domain contribute to the robustness of CENP-C recruitment to kinetochores [151,165]. 

In vitro, CENP-C and CENP-N show relatively modest selectivity for CENP-A over H3, with differences in dissociation constant of between 5- and 10-fold [110,151]. It is unlikely that these differences, in the absence of other factors, account for the exquisite selectivity of kinetochore targeting of these proteins to CENP-A nucleosomes, which are greatly outnumbered by H3 nucleosomes at centromeres and in the rest of the genome [169]. Dimerization of CENP-C through its C-terminal cupin-like domain (Figure 3C) suggests a role for multi-valency as a source of additional selectivity for the interaction of CENP-C with centromeric CENP-A nucleosomes [79]. A second source of selectivity may derive from the interaction, within the CCAN of CENP-C and CENP-N, which recognize distinct features of the CENP-A nucleosome [110,151]. Post-translational modifications of histones have also been implicated as a potential factor in the selective recognition of CENP-A nucleosomes [170,171].

In addition to CENP-C and CENP-N, the CENP-HIKM complex (Figure 2D) also contributes to CENP-A binding affinity, but this complex interacts equally well with CENP-A and H3 nucleosomes, and with linear DNA [110]. Importantly, however, CENP-C, CENP-HIKM, and CENP-LN interact in a tight 7-subunit complex, the CENP-CHIKMLN complex [110], whose stability builds on multiple interactions of its subunits, including direct interactions of CENP-HIKM or CENP-LN with CENP-C [2,108,109,110,166].

A comprehensive view of the structural organization of the CENP-CHIKMLN complex is currently missing. Crystal structures of the CENP-LN complex of *S. cerevisiae* and of human CENP-M have been obtained and negative-stain single particle EM reconstructions have been generated for CENP-HIKM [108,111]. CENP-M is structurally and evolutionary related to Ras family small GTPases. It has lost all signature motifs previously implicated in GTP binding and hydrolysis by small GTPases, and is therefore considered a pseudo-GTPase [111]. Biochemical reconstitution demonstrated that CENP-M is required to stabilize CENP-I, predicted to have a α-solenoid fold of β-karyopherins [111]. No CENP-M ortholog has been identified in *S. cerevisiae* whereas CENP-H, -I, and -K all have orthologs in this organism (Figure 2A,D) [88].

### 3.4. The CENP-TWSX Complex

The CENP-TW and CENP-SX subcomplexes (Figure 2D) associate to form the tetrameric CENP-TWSX complex. All four subunits in this tetrameric complex possess histone fold domains. CENP-T contains additional N-terminal sequences, whose function in kinetochore assembly is discussed in Section 4. While CENP-TW and CENP-SX form stable entities in isolation and can have distinct biological functions [172,173,174], the tetrameric CENP-TWSX assembly was proposed to form a nucleosome-like structure flanking the CENP-A nucleosome at centromeres [175]. However, rigorous structural and functional evidence for this hypothesis is lacking. In vitro, the CENP-TWSX complex induces positive DNA supercoiling, contrarily to H3 and CENP-A nucleosomes (and to the isolated CENP-TW and CENP-SX complexes), which induce negative supercoiling [176]. When incubated with in vitro reconstituted H3 or CENP-A di-nucleosomes and visualized by negative-stain EM, two tetramers of CENP-TWSX bound preferentially to the ~100 bp inter-nucleosome linker DNA rather than nucleosome-bound DNA, but the limited resolution did not allow discriminating whether the two CENP-TWSX tetramers formed a nucleosome-like structure on the linker DNA; in addition, nuclease cleavage did not identify the pattern normally observed with canonical nucleosomes [176].

DNA binding by CENP-TWSX requires the histone fold domains of CENP-T and CENP-W [100,175,176]. No evidence of DNA sequence selectivity for these domains has been reported. In contrast to CENP-A, the pool of CENP-TW turns over relatively rapidly at centromeres, and with a cell cycle-regulated pattern, with centromere incorporation in late S-phase and G2 [177]. Incorporation of CENP-TW at the kinetochore has not been shown to require histone chaperones. Rather, in both humans and yeast, kinetochore recruitment of CENP-T^Cnn1^ appears to depend, in addition to DNA binding, on a direct interaction with other CCAN subunits, and in particular with the CENP-HIKM complex [78,111,113,178]. CENP-T recruitment is also affected by the N-terminal tail of CENP-A, in both fission yeast and humans [70,179], although the biochemical basis for this effect is to date unclear. Furthermore, ablation of either CENP-TW or CENP-SX has a distinct effect on outer kinetochore stability [100,101,178]. These observations detract from the hypothesis of a nucleosome-like structure flanking the CENP-A nucleosome, and rather suggest that CENP-TW binds DNA weakly and requires concomitant binding to other CCAN subunits at the inner kinetochore for its recruitment.

### 3.5. The CENP-OPQRU Complex

CENP-O, -P, -Q, -R, and -U (Figure 2D) associate into a complex [109,180]. Recruitment of CENP-OPQRU to the kinetochore requires CENP-CHIKMLN [96,97]. Loss of this complex does not affect localization of other inner kinetochore components and the functional importance of this complex at vertebrate kinetochores appears to vary in different systems [109,181,182]. A role in chromosome congression, at least partly operating through microtubule-binding sites in the CENP-Q and CENP-U subunits, as well as through recruitment of the microtubule motor CENP-E to kinetochores, has been reported [182,183,184]. Furthermore, CENP-U has been implicated in kinetochore recruitment of Polo-like kinase 1 (Plk1), an important regulator of kinetochore-microtubule attachments [185,186]. Thus, this complex is relatively peripheral in the organization of the CCAN in vertebrates and may contribute to chromosome segregation via recruitment of motor and kinase activities. 

In budding yeast, where the CENP-O/P/Q/U/R–related complex is known as the COMA complex (for Ctf19, Okp1, Ame1 and Mcm21), two of the subunits, Ctf19 and Mcm21 (homologous to CENP-P and CENP-O, respectively), are non-essential for viability but are required for accurate segregation [187]. These subunits harbor RWD domains related to those observed in the Spc24/Spc25 subunits of the Ndc80 complex and the Knl1 C-terminus [116]. Deletion of Ctf19 or Mcm21 disrupts proper replication timing and cohesin complex accumulation in the pericentromeric region [188,189,190]. Interestingly, the other two subunits, Ame1 and Okp1 (homologous to CENP-U and CENP-Q, respectively) are essential for viability and Ame1 has been shown to directly interact with the Mis12 complex of the KMN network via a motif whose selective mutation is lethal [191]. These results suggest a potentially more significant role for the COMA complex in inner-outer kinetochore linkage in budding yeast than has been observed for the CENP-O/P/Q/U/R complex in vertebrates. CENP-Q is essential in mouse embryonic stem cells but not in mouse fibroblasts [181]; whether this difference in phenotype arises from a differential role in kinetochore assembly is not known. It will be important to elucidate the functions of this peripheral CCAN complex and determine the reasons for distinct effects of its loss in different systems and in different contexts within the same species.

### 3.6. CENP-B

CENP-B, the only specific DNA binding protein at mammalian centromeres, binds to the conserved 17-bp CENP-B-box, many copies of which are disseminated in centromeric α-satellite DNA repeats [192] (Figure 2E). CENP-B shares sequence homology with transposases encoded by the *pogo* family of DNA transposons [193] and appears to have arisen from them. A role of CENP-B in centromere stability has been questioned because of its limited conservation (CENP-B like proteins are thought to have arisen from transposases independently in fungi, insects and mammals), because CENP-B boxes are absent from neocentromeres, and because there are chromosomes (such as the human Y chromosome) that lack CENP-B boxes altogether. Furthermore, deletion of CENP-B in mice does not affect viability [194,195,196]. Nonetheless, a role for CENP-B in centromere stability is suggested by its requirement for the de novo establishment of centromeres on human artificial chromosomes built using centromere-enriched satellite DNA [197,198], and from the fact that its deletion increases chromosome instability [199,200]. Several recent and older observations support a role of CENP-B in the stabilization of centromere structure. For instance, CENP-B appears to contribute to the phasing of CENP-A nucleosomes on centromeric DNA and to the typical unwrapping of its nucleosomal termini [146], and it increases the stability of reconstituted CENP-A nucleosomes [201], likely through a direct interaction with the N-terminal region of CENP-A [199]. Furthermore, CENP-B binds directly to CENP-C, supporting a second pathway of CENP-C recruitment in addition to that based on the interaction of CENP-C with the CENP-A C-terminal tail [159,199]. These interactions of CENP-B may be largely redundant with other stabilizing interactions at centromeres, but their importance is exposed following perturbation of normal CENP-A function [199].

### 3.7. Summary

This section illustrates the important concept that inner kinetochores are built by evolutionary conserved interactions of CCAN subunits with the CENP-A nucleosome. In line with the theory that centromere identity is determined epigenetically in most organisms (the notable exception being budding yeasts, where a specific sequence is recognized by the CBF3 complex to direct CENP-A loading), none of these interactions, appears to require specific DNA sequences, except for those made by CENP-B, which is not conserved even throughout vertebrates and is not essential but may contribute to kinetochore stability when present. The evolutionary presence/absence and phenotypic effect of CCAN subunit inhibitions, with the possible exception of CENP-C, are also surprisingly variable, with kinetochores of well-studied models such as *C. elegans* and *D. melanogaster* lacking the entire repertoire except for CENP-C [62,88,89,133]. These greatly simplified kinetochores appear to entirely rely on CENP-C being a linker between the CENP-A nucleosome and the outer kinetochore, as discussed in Section 5. This variation raises intriguing questions about the functional roles of this large group of inner kinetochore proteins that will be important to address in future studies.

## 4. The Outer Kinetochore

The outer kinetochore is the (main) platform for end-on microtubule binding by the kinetochore and responsible for transducing the force generated by depolymerizing microtubules to move chromosomes. The core of the outer kinetochore is a 10-subunit protein assembly known as KMN (for Knl1 complex, Mis12 complex, Ndc80 complex, described in Figure 2) [115,202,203,204,205,206,207,208,209,210,211,212]. The three sub-complexes (Knl1, Mis12, and Ndc80) exercise clearly distinct functions as summarized below.

### 4.1. The Ndc80 Complex

**Figure 4:**
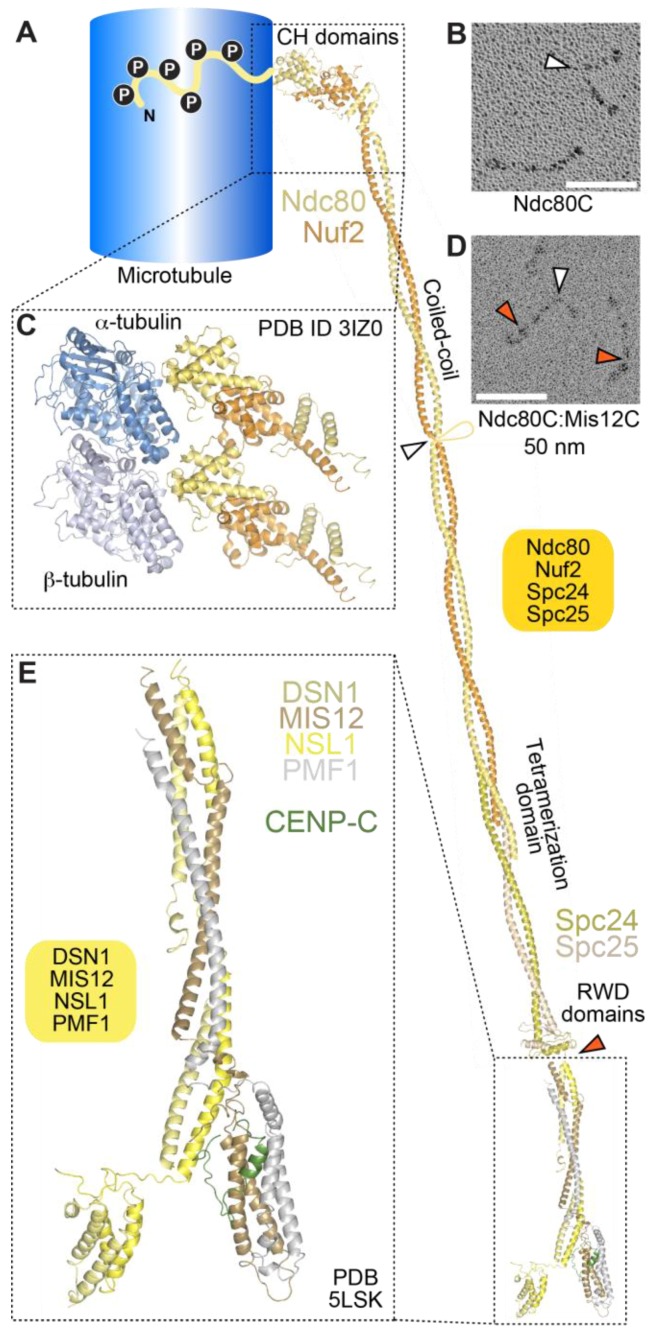
The NDC80 and MIS12 complexes of the KMN network. (**A**) The NDC80 complex is highly elongated and interacts with microtubules via calponin-homology (CH) domains in the N-terminal regions of NDC80 and NUF2. A basic N-terminal tail preceding the NDC80 CH domain (depicted as unstructured) is subject to Aurora kinase phosphorylation (*Ps in black circle*s) and regulates microtubule binding. A long coiled-coil, interrupted by a loop (*white arrowhead*) terminates in a tetramerization domain with SPC24 and SPC25. The latter start with coiled-coils and terminate with RWD domains (*red arrowhead*), which interact with the MIS12 complex; (**B**) Rotary shadowing electron microscopy of the NDC80 complex, showing its characteristic dumbbell shape, and an overall length of ~65 nm. Images in (**B**,**D**) courtesy of Dr. Pim Huis in ‘t Veld, Max Planck Institute of Molecular Physiology, Dortmund (Germany) [239]; (**C**) Model from cryo-EM studies of the Ndc80^Bonsai^ complex bound to the microtubule lattice. Only a single α-tubulin:β-tubulin dimer is shown, with two Ndc80^Bonsai^ complexes bound via the toe region; (**D**) Complexes of the NDC80C and MIS12C are ~85 nm in length; (**E**) Structural organization of the MIS12 complex bound to the N-terminal region of CENP-C [241]. All structures shown are for the human complexes.

The 4-subunit Ndc80 complex is the primary microtubule receptor at the kinetochore [209,213]. Its four subunits contain large segments of coiled-coil, flanked by globular domains (Figure 4A). The complex is dumbbell-shaped and has a long axis of approximately 55 to 60 nm (Figure 4A,B). Microtubule-binding, mediated by the N-terminal regions of the Ndc80 and Nuf2 subunits, and kinetochore-targeting, mediated by the C-terminal regions of the Spc24 and Spc25 subunits, occupy opposite ends of the complex [214,215,216,217,218].

Crystal structures of isolated globular domains of Ndc80 complex subunits, and of engineered Ndc80 complexes lacking most of the coiled-coil (named Ndc80^Bonsai^ and Ndc80^Dwarf^), revealed that the microtubule-binding region of the Ndc80 complex consists of a pair of tightly packed calponin-homology (CH) domains in the Nuf2 and Ndc80 (also called Hec1) subunits [216,218,219]. The latter are structural paralogs, whose overall domain organization, an N-terminal CH domain adjoined by a coiled-coil segment, is also found in three Intraflagellar Transport (IFT) complex B subunits, IFT81, IFT57, and CLUAP1 [220]. 

Visualization by cryo-electron microscopy of Ndc80^Bonsai^ bound to microtubules demonstrated a direct interaction of the CH domain of the Ndc80 subunit with the microtubule lattice with a spacing of 4 nm along each protofilament, indicative of interactions of the CH domain with both tubulin monomers [209,221,222] (Figure 4C). The interaction engages a region of the Ndc80 CH domain, designated toe, which gathers several positively charged residues previously shown to mediate high-affinity microtubule binding, and which has been proposed to act as a conformational sensor for straight protofilaments [221,223].

In addition to the CH domains, an ~80-residue, highly basic and structurally disordered N-terminal tail of the Ndc80 subunit has been functionally implicated in the Ndc80-microtubule interaction in vitro and in cells [213,216,218,224,225,226] (Figure 4A). The N-terminal tail may contain two distinct functional segments. One segment, running from residues 47–68 (of human Ndc80), has been implicated directly in microtubule binding through an interaction with E-hooks (the negatively charged C-terminal tails of α- and β-tubulin) of tubulin protomers in the adjacent protofilament [222]. Another segment, preceding the E-hook binding region, has been implicated in inter-Ndc80 complex interactions along the same protofilament [221,222]. Collectively, these interactions may be responsible for the ability of the Ndc80 complex to form clusters on the microtubule lattice [218,221,222], and suggest that binding of Ndc80 complexes to microtubules may be cooperative. As explained more thoroughly below, however, a microtubule-binding site in the kinetochore engages a relatively small number of Ndc80 complexes (probably 6 to 10) in an end-on configuration. Whether Ndc80 complexes can interact inter-molecularly in this setting, and whether their binding to microtubules is cooperative, remains controversial [227,228,229]. We additionally note that deletions of the Ndc80 tail in *S. cerevisiae* and in *C. elegans* do not exhibit the severe phenotypic consequences expected for a major defect in kinetochore-microtubule interactions [230,231,232]; in contrast, mutations in conserved CH domain residues have severe consequences in all systems where they have been analyzed. Thus, the precise role of the N-terminal tail of Ndc80 in kinetochore-microtubule interactions remains an important question for future investigation.

While the mechanism of the interaction of Ndc80 with microtubules and with itself requires further investigation, it is clear that the N-terminal tail of Ndc80 regulates microtubule binding. Aurora B kinase, a major regulator of kinetochore-microtubule attachment [14], phosphorylates up to nine sites in the human Ndc80 N-terminal tail [213,216,218,221,222,224,225,226,229,233]. Phosphorylation neutralizes the intrinsic positive charge of the Ndc80 N-terminal domain, greatly decreasing the binding affinity of the Ndc80 complex for microtubules in vitro [209,216,218,225,226]. The N-terminal tail of *C. elegans* Ndc80 is also the target of regulation by a protein complex that recruits and activates the dynein motor at kinetochores [232]; see Section 5.3 below.

### 4.2. The Mis12 and Knl1 Complexes

The Mis12 complex is an interaction hub that promotes KMN assembly through its binding sites for both the Ndc80 complex and the Knl1 complex, and that connects the KMN with the inner kinetochore through interactions with CENP-C and CENP-T. Previous low-resolution negative stain EM analyses depicted the Mis12 complex (known as MIND complex in *S. cerevisiae*) as a ~20 nm rod [234,235,236,237]. When Mis12 and Ndc80 are combined and their structure is examined by negative stain or rotary shadowing EM, they appear as ~90-nm particles, indicating that they interact ‘in series’ [238,239] (Figure 4D). Recent crystal structures of the human and yeast complexes demonstrate that the four subunits of the Mis12 complex are structural paralogs with high helical content (Figure 4E). They pair in Dsn1:Nsl1 and Mis12:Pmf1 sub-complexes, that meet in a central stalk domain. The N- and C-termini of all four subunits cluster at opposite ends of the rod [240,241]. Linear motifs near the C-termini of the Nsl1 and Dsn1 subunits of the Mis12 complex, invisible in the crystal structure, provide binding sites for the RWD domains in the C-terminal region of the Spc24 and Spc25 subunits of the Ndc80 complex [234,240,241,242].

**Figure 5:**
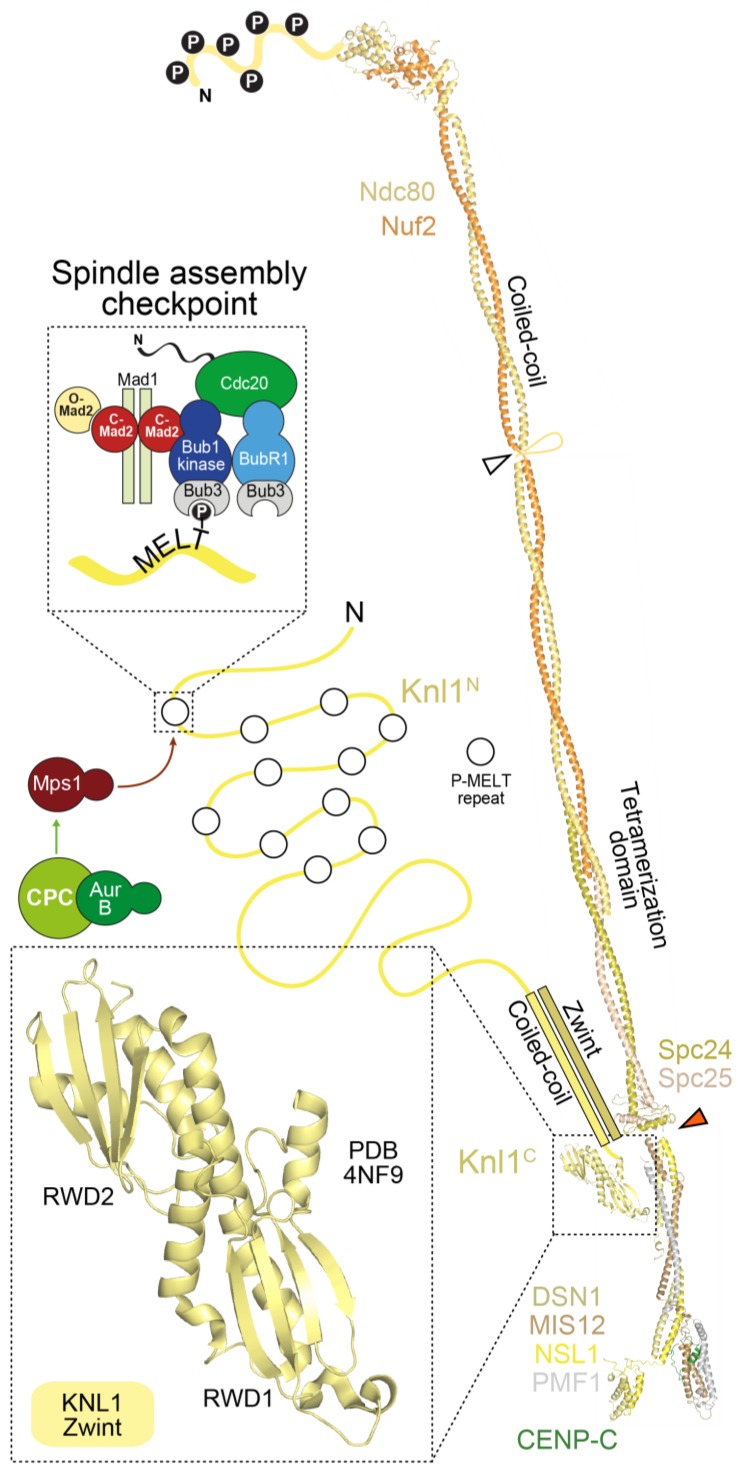
Orchestration of the spindle assembly checkpoint (SAC) by the KMN network SAC components are recruited via the Knl1 subunit that is 2316 residues in humans and is largely disordered. Exceptions are a predicted coiled-coil around residues 1850–2100, and the C-terminal tandem RWD domains, whose crystal structure is shown [235]. The RWD region of Knl1 binds directly to the MIS12 complex [234,235]. The N-terminal half of Knl1 contains multiple MELT repeats (Met-Glu-Leu-Thr) that are targeted by Mps1 kinase (which in turn requires Aurora B kinase to become activated). Each MELT repeat has the potential to assemble active SAC complexes that signal lack of microtubule attachment and arrest the cell cycle in mitosis.

The stalk of the Mis12 complex, together with a ~20-residue C-terminal motif in Nsl1, also provides a binding site for Knl1, the largest outer kinetochore subunit (2316 residues in humans) (Figure 5). With the exception of the last ~500 residues, Knl1 is largely intrinsically disordered, and contains an array of protein docking motifs, including a canonical binding site for the PP1 phosphatase very near the N-terminus, and multiple Met-Glu-Leu-Thr (MELT) repeats, identified to act, after phosphorylation by the Mps1 kinase on the conserved Thr residue, as docking sites for the SAC protein complex Bub1:Bub3 [15,17] (Figure 5). The Knl1 C-terminal region, on the other hand, consists of a predicted coiled-coil followed by tandem RWD domains, and is therefore structurally related to Spc24, Spc25, CENP-O, and CENP-P, suggesting a common evolutionary origin of these proteins [116,235]. The RWD domain mediates a direct interaction of Knl1 with the Mis12 complex, whereas Zwint binds to a more extended domain additionally comprising the coiled-coil region [234,235,241]. 

With some variations, the description of the outer kinetochore in the previous paragraphs applies to yeast and human kinetochores alike. In addition, in the case of the outer kinetochore, exceptions have emerged in the course of evolution. In *D. melanogaster*, for instance, no ortholog of Dsn1 or Zwint was identified, whereas two closely related and functionally redundant paralogs of Pmf1^Nnf1^ (Nnf1a and Nnf1b) exist [243,244,245,246]. Furthermore, the unconventional SNARE family member Snap29 was recently shown to localize to kinetochores and to be required for KMN assembly in this organism [247]. In biochemical reconstitutions, the *Drosophila* Mis12 complex is highly stable in the absence of Dsn1 (unlike the human and yeast complexes) [245,246]. Significant adaptation of the Nsl1 sequence at residues implicated in Dsn1 binding by the structures of the human and yeast Mis12 complex explain this result (not shown). In *C. elegans*, where the KMN components are all present and were shown to self-associate in biochemical reconstitutions, there are some notable sequence variations, e.g., *C. elegans* Knl1 lacks the tandem RWD domains at the C-terminus and the RWD domains of Spc24 and Spc25 appear severely diminished. Knl1 family proteins also exhibit widespread and recurrent evolution of repeats in their N-terminal region [248]. The reasons for these variations are currently unclear. Nonetheless, relative to the CCAN, KMN is broadly conserved, likely reflecting its essential involvement in microtubule attachment and scaffolding of the SAC.

### 4.3. Complexity of the Kinetochore Microtubule Interfaces

Understanding the role of Ndc80 complex in the generation of dynamic load-bearing attachments during chromosome congression and segregation is a primary goal of current research (see review by Asbury and colleagues, reference [30]). Classic in vitro studies demonstrated that kinetochores can hold on to a depolymerizing microtubule end [249] and, more significantly, a depolymerizing microtubule generates significant force that is capable of moving chromosomes bound via their kinetochores [250,251]. These studies inspired efforts to analyze if the Ndc80 complex, the widely conserved outer kinetochore-localized microtubule-binding complex, acts as a coupler that is able to harness the force generated by a depolymerizing microtubule end. When immobilized on beads at sufficiently high concentrations, the Ndc80 complex is sufficient to create load-bearing attachments to depolymerizing microtubules [29,227,252]. Individual, soluble Ndc80 complexes, on the other hand, are unable to track depolymerizing microtubule ends [253]. These experiments suggest that clustering on beads enables the establishment of multiple microtubule attachments, and that the latter are required for microtubule plus-end tracking during depolymerization. Clustering of multiple Ndc80 complexes at kinetochores is likely to achieve a similar effect. 

It is important to note, however, that Ndc80 is not the sole player in linking kinetochores to microtubules. Additional microtubule-binding proteins and motors identified there include the SKA and Dam1 complexes, Kif18a, MCAK, SKAP:Astrin, XMAP215/CH-TOG, CENP-E, CENP-F, and Dynein [9,254]. The functions of these proteins at kinetochores are discussed in the essays from Maiato, Lampson and Grishchuk, and Asbury and colleagues ([14,30,255]), and here we limit the discussion to a brief account of the SKA and the Dam1 complexes, two sequence and structurally unrelated microtubule binders with complementary phylogenetic distributions that have emerged as playing a fundamental role in microtubule coupling at kinetochores [256,257,258,259]. The human SKA complex and the budding yeast Dam1 complex can track dynamic microtubules, and interact with their cognate Ndc80 complex specifically when bound to microtubules [253,260,261,262,263,264,265]. The Aurora B kinase phosphorylates the SKA and Dam1 complexes to reduce their binding affinity for kinetochores [264,265,266,267], in line with a regulatory scheme that identifies the Aurora B kinase as a negative regulator of the strength of the attachment of kinetochores to microtubules, and as a crucial actor in the correction of improper kinetochore-microtubule attachments (see reviews by Lampson and Grishchuk and by Asbury and colleagues [14,30]). In a recent twist, the SKA complex was also shown to stimulate Aurora B activity [268].

Structural and biochemical work on the SKA complex has started to elucidate its organization and mechanism of action [253,269,270,271]. The SKA complex is a trimer of the Ska1, Ska2, and Ska3 subunits. It is ‘W’ shaped, and consists of dimers of triple helical bundles of the three subunits [269]. The C-terminal domain of the Ska1 subunit contains a winged-helix motif that interacts with surface-exposed regions of tubulin that are insensitive to microtubule curvature, while the unstructured C-terminal region of Ska3 facilitates the interaction of Ska1 with microtubules [271]. The Dam1 complex is a heterodecamer [272,273], and individual heterodecamers assemble into rings that encircle the microtubule surface [274,275,276,277]. Both the SKA and Dam1 complexes are dependent on the Ndc80 complex for their kinetochore localization in cells and enhance the microtubule coupling ability of the Ndc80 complex in vitro. These findings suggest that the concerted action of Ndc80 and SKA/Dam1 complexes underlies the load-bearing attachments made at kinetochores but the detailed mechanistic basis for their concerted action remains to be elucidated.

## 5. Linkages between the Inner and the Outer Kinetochore

### 5.1. Two Mechanisms Link Inner and Outer Kinetochores

**Figure 6:**
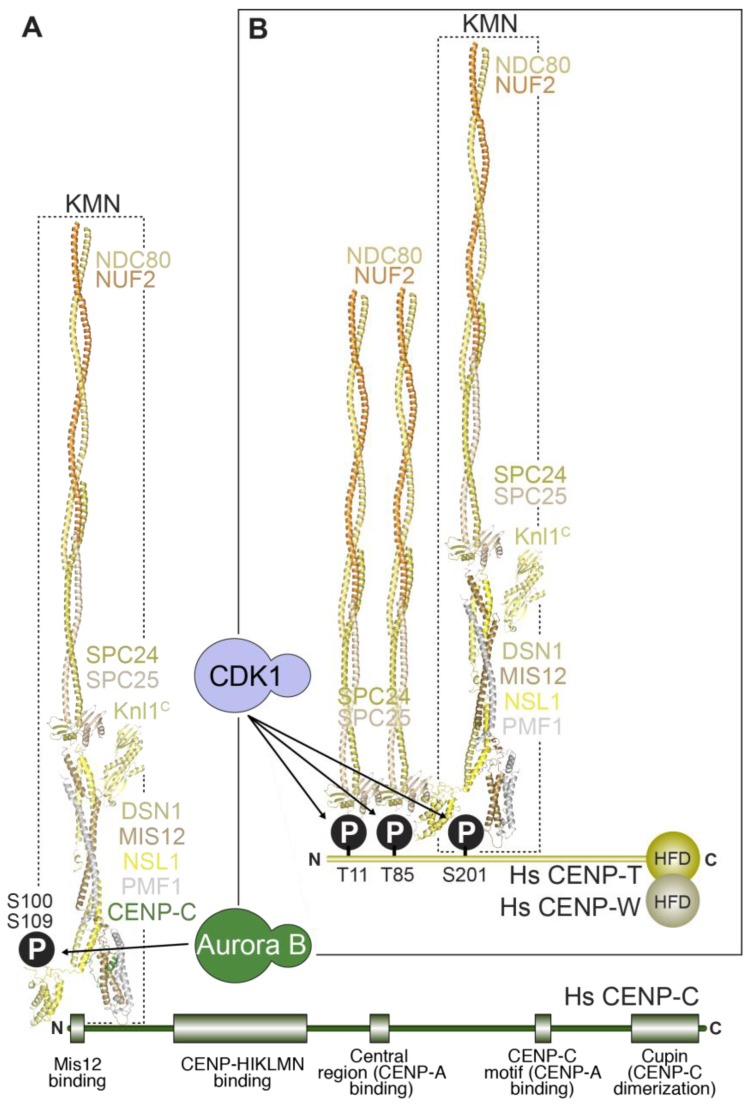
Linkages between the inner and outer kinetochore. Structures of a portion of the NDC80 complex (the N-terminal globular domains of NDC80 and NUF2 are not shown), the MIS12 complex and the C-terminal kinetochore-targeting domain of KNL1 are used to depict a KMN particle in humans. (**A**) The first linkage is formed by the interaction of a KMN particle with the N-terminal region of CENP-C. This interaction is enhanced by Aurora B phosphorylation of residues (S100 and S109) in the N-terminal region of the DSN1 subunit of the MIS12 complex; (**B**) The second linkage involves the interaction of up to two NDC80 complexes with two CDK1-phosphorylated residues (T11 & T85) in the N-terminal region of CENP-T, as well as of a second entire KMN recruited via a CDK1-dependent interaction of the MIS12 complex with S201 of CENP-T [239]. In vitro, CENP-C and CENP-T bind to the MIS12 complex within the KMN network competitively, implying that they cannot be bound to the same KMN [239]. All structures shown are for the human complexes.

The outer kinetochore is linked to the inner kinetochore via two different mechanisms (Figure 6). In the first mechanism, CENP-C directly binds to the Mis12 complex [165,238,240,241,245,278,279], which in turn binds to the Ndc80 complex and Knl1. In the second mechanism, the RWD domains in the Spc24 and Spc25 subunits of the Ndc80 complex directly interact with the intrinsically disordered N-terminal extension of CENP-T [75,113,239,242,279,280,281,282]. We summarize below the detailed understanding of these two mechanisms in yeast and vertebrates and their relative importance in outer kinetochore assembly in different systems.

CENP-C (and its yeast homolog Mif2) binds directly to the Mis12 complex through an ~45-residue N-terminal motif, an interaction captured in the recent co-crystal structures of the yeast and human Mis12^MIND^ complexes discussed in the previous section [240,241] (Figure 4E). In *S. cerevisiae*, the 4-subunit COMA complex (Ctf19:Okp1:Mcm21:Ame1), which is part of the yeast CCAN^Ctf19^ complex and whose subunits are related to those in the CENP-OPQRU complex, helps reinforce the interaction of CENP-C^Mif2^ with Mis12^MIND^ [191]. In both yeast and humans, Aurora B kinase regulates the Mis12^MIND^:CENP-C^Mif2^ interaction by phosphorylation of two serine residues (Ser100 and Ser109 in humans) that reside in closely spaced, positively charged motifs in a disordered region of Dsn1 [280,283,284,285,286,287]. Aurora B phosphorylation of Dsn1 increases the binding affinity of CENP-C for the Mis12 complex by approximately two orders of magnitude, through relief of a competitive inhibitory mechanism in which the unphosphorylated Dsn1 region binds and masks in an intra-Mis12-complex manner the CENP-C binding region [240,241]. The significance of this regulation, which likely requires the presence of Aurora B kinase activity at centromeres, may be to stabilize the CENP-C:Mis12 complex interaction exclusively in the proximity of kinetochores.

The RWD domains in the Spc24 and Spc25 subunits of the Ndc80 complex interact directly with two related, short sequence motifs in the first 100 residues of the intrinsically disordered N-terminal extension of CENP-T (consisting of approximately 450 residues in humans) [75,113,239,242,279,280,281,282]. In humans, this interaction requires CDK phosphorylation of the CENP-T motifs, but the equivalent interaction of Spc24:Spc25 with CENP-T^Cnn1^ in *S. cerevisiae* may not require phosphorylation [242,279,281]. At least in vitro, the two Ndc80 complex-binding motifs of CENP-T can be occupied concomitantly, suggesting that this mechanism can recruit up to two Ndc80 complexes per CENP-T molecule [113,239]. The motifs on CENP-T are closely related to the Spc24:Spc25-binding motif in Dsn1 (discussed in the previous section) [242]. Not surprisingly, therefore, recent crystal structures demonstrated that the Dsn1 and CENP-T motifs bind Spc24:Spc25 through a largely similar mechanism [240]. 

In an interesting recent twist, it was realized that CENP-T also contributes to kinetochore recruitment of the Mis12 complex [239,280,288]. This is promoted by a direct interaction of the Mis12 complex with a distinct, non-canonical CDK phosphorylation site on human CENP-T, Ser201 [239]. Whether this interaction is conserved in *S. cerevisiae* is currently unknown. Thus, at human kinetochores, a single N-terminal tail of CENP-T can, after appropriate phosphorylation, promote the localization of up to three Ndc80 complexes, two through a direct interaction, and one indirectly through the Mis12 complex (Figure 6). These biochemical data are consistent with analysis in human cells, where CENP-T depletion reduces Ndc80 complex localization at kinetochores to a third of that in controls, without affecting CENP-C localization (See Section 5.2 below).

The presence of two mechanisms for linking the outer and inner kinetochore raises the question why these two linkages are needed and whether they are widely employed. In all systems tested, CENP-C inhibition leads to severe defects and lethality (with the exception of *S. pombe*, where a suppressor mutation can improve growth of a CENP-C null mutant that is extremely sick and missegregates chromosomes at high frequency [166]. Depletion or deletion of CENP-T results in extensive outer kinetochore assembly and chromosome alignment defects in chicken and human cells [100,176,279,280,287,288]. In addition, chicken CENP-T can generate ectopic microtubule attachment sites that support chromosome segregation in the absence of CENP-C [75], and a chimeric construct in which the N-terminal region of CENP-T replaced the entire N-terminal domain of CENP-C appeared to support chromosome segregation [288]. Surprisingly, however, a deletion mutant of the CENP-T ortholog Cnn1 in *S. cerevisiae* is viable and the absence of CENP-T does not significantly reduce the amount of Ndc80 recruited to kinetochores in this system [242,282,289]. In addition, in *D. melanogaster* and *C. elegans*, which lack all CCAN subunits with the exception of CENP-C (see Section 3), the interaction of CENP-C with the Mis12 complex is likely the only linkage between the inner and outer kinetochore. Consistent with this notion, a tight CENP-C interaction with the Mis12 complex has been observed in biochemical reconstitutions of the *D. melanogaster* outer kinetochore [245,246]. Thus, from the analysis in different models to date, it appears that the CENP-C–outer kinetochore linkage is more commonly employed, although in the vertebrate species analyzed to date the CENP-T linkage makes the more dominant contribution to Ndc80 complex recruitment. Interestingly, the CENP-T ortholog in *S. pombe* (Cnp20) unlike Cnn1 in *S. cerevisiae*, is essential for viability [166]. Additional work on *S. pombe* CENP-T is needed to address whether its essential function relates to outer kinetochore assembly. More broadly, asking precisely why two types of linkages have evolved to link the inner and outer kinetochore and asking whether there is a functional specialization of these linkages are important questions for future studies.

In summary, the plan of kinetochore assembly from the chromatin layer to the outer kinetochore has been now delineated in significant detail. Crucial features of this assembly plan include: (1) Recruitment of all kinetochore proteins ultimately depends on specific interactions with CENP-A. CENP-C, CENP-N, and CENP-T, which have been implicated as the proteins at the base of the kinetochore, require CENP-A for their localization; (2) Both CENP-C and CENP-N bind directly to the CENP-A nucleosome; (3) CENP-T does not bind directly to the CENP-A nucleosome, but appears to recognize a combination of the CENP-HIKM complex (which interacts directly with CENP-C and CENP-NL) and naked DNA, possibly in a linker region neighboring the CENP-A nucleosome; (4) CENP-C creates a direct linkage between CENP-A (and its associated CCAN subunits) and the KMN network, binding concomitantly to both, and acting in analogy to a ‘blueprint’ to order kinetochore assembly; (5) CENP-T plays an analogous bridging function, and its N-terminal region can even replace the N-terminal region of CENP-C involved in Mis12 binding in an engineered context. The latter observation suggests that CENP-C and CENP-T might be distantly related in evolution. 

These principles, which summarize a vast body of literature, were recently implemented in the biochemical reconstitution of a 21-subunit kinetochore particle containing the CENP-A nucleosome, the CENP-CHIKMLN complex, and the KMN network [110]. The reconstituted complex was shown to be sufficient to associate the CENP-A nucleosome with microtubules in vitro, demonstrating that its components can create a linkage between DNA associated with CENP-A and microtubules. Lacking from the reconstitution were the CENP-TW (and CENP-SX) complex and the CENP-OPQRU complex. The latter binds directly and with high affinity to the CENP-CHIKMLN complex, whereas incorporation of the former might require, as suggested above, a more complex chromatin template than a single CENP-A nucleosome.

**Figure 7:**
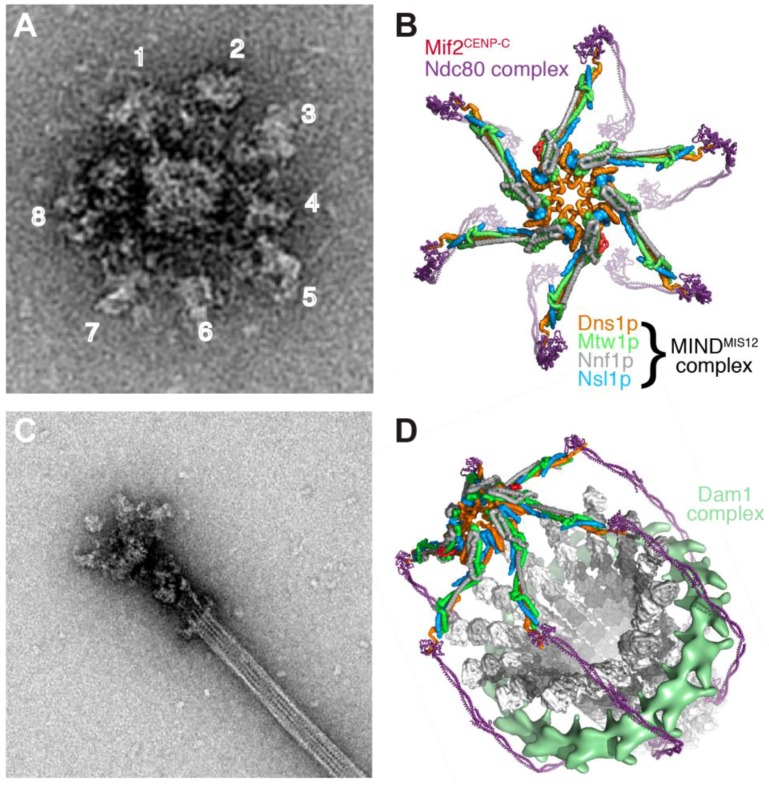
Images and structural model of budding yeast kinetochore particles. (**A**) Negative stain electron micrographs showing a kinetochore particle isolated from *S. cerevisiae* [291]. Images in this panel and in **C** courtesy of Sue Biggins and Tamir Gonen; (**B**) A rendered image with possible molecular interpretation of the negatively stained particles showing MIND^MIS12^:CENP-C complexes departing from a central “hub” and connecting with Ndc80 complexes. Image reproduced with permission from reference [240]; (**C**) Negative stain electron micrographs showing a *S. cerevisiae* kinetochore particle bound to the end of a taxol-stabilized microtubule [291]. Image courtesy of Sue Biggins and Tamir Gonen; (**D**) The structure in (**B**) is shown to surround the microtubule in end-on configuration. The Dam1 complex stabilizes the arrangement by surrounding the microtubule. Image reproduced with permission from reference [240].

A reconstitution approach also has the potential to fully define the CEN DNA-based kinetochore in *S. cerevisiae* that is built on a single well-positioned CENP-A nucleosome and binds to a single microtubule. Work on isolated kinetochore particles purified from *S. cerevisiae* has begun to illustrate the overall structural organization of this unit kinetochore and its microtubule binding modes [290,291] (Figure 7). When imaged by negative stain EM, the kinetochore particles had a central core of ~37 nm diameter, and were radially surrounded by 5 to 7 globular domains with ~21 nm diameter. When bound to microtubules, the particles appeared to contain a 50-nm ring structure surrounding the microtubule (likely the Dam1 complex), linked through a fibrous network (likely the Ndc80 complex) to the globular region. Two sites of microtubule attachment were visible, one coinciding with the ring structure and one at the junction of the fibrous structure with the globular region [291]. Continued analysis of yeast kinetochore particles and of human kinetochore reconstitutions of the type described recently should yield detailed insight into the structure and microtubule interaction properties of a unit kinetochore module in the foreseeable future. 

### 5.2. Stoichiometry of Kinetochore Subunits

**Figure 8:**
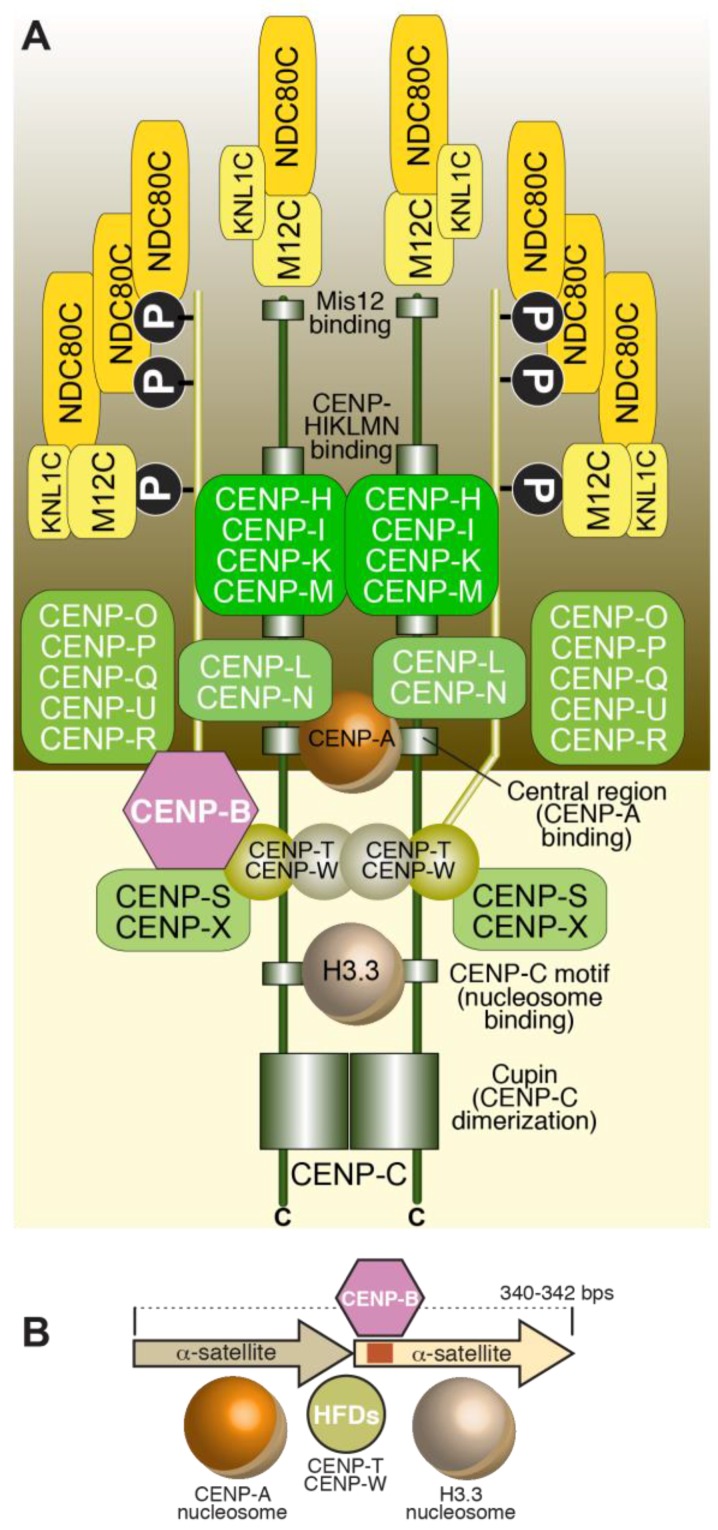
A model for the assembly unit of the human kinetochore. The kinetochore assembly unit is depicted as being organized on a CENP-A-H3.3 dinucleosome. (**A**) A primary determinant of the stoichiometry of kinetochore subunits is the valency of the CENP-A nucleosome, which confers the potential to interact with two CCAN complexes, as shown in work of in vitro reconstitution of kinetochore assembly [110]. Because CENP-C and CENP-T each carries a full KMN network, and CENP-T additionally carries two NDC80 complexes, there are four MIS12 and KNL1 complexes per CENP-A nucleosome, and up to 8 NDC80 complexes. CENP-C has the potential to interact with two nucleosomes (see Figure 3), with one of them (the one bound to the CENP-C central region) is a CENP-A nucleosome permanently marked by interactions with stably bound CCAN subunits. As clarified in Figure 9, we speculate that the identity of the second nucleosome, which binds to the CENP-C motif, varies during the cell cycle, alternating between CENP-A and H3.3. The C-terminal dimerization domain of CENP-C might “seal” this design. During mitosis, the second nucleosome is an H3.3 nucleosome; (**B**) This speculative design is compatible with the existence of the tandem α-satellite structures already discussed in Figure 2. CENP-TW is proposed to bind in the inter-nucleosomal linker region, near the CENP-B box.

Estimates of the stoichiometry of human kinetochore composition were recently obtained through distinct experimental efforts, including biochemical reconstitution combined with analytical ultracentrifugation, and measurements of fluorescence intensity ratios in cells [110,288]. Biochemical reconstitution suggests that there are two CCAN complexes per CENP-A nucleosome [110]. As explained above, both CENP-C and CENP-T interact directly with the Mis12 complex, but their binding is mutually exclusive, implying that each of the two CCAN subunits has the potential to recruit the Mis12 complex independently [239]. Thus, if two copies of CENP-C and CENP-T associate with a CENP-A nucleosome, and each of them recruits Mis12, four Mis12 complexes will associated with the CENP-A nucleosome. Because each Mis12 complex also carries tightly bound Ndc80 and Knl1 complexes, as predicted by biochemical reconstitution experiments, at least four of each should be present. Furthermore, each CENP-T can also directly recruit up to two additional Ndc80 complexes, depending on the degree of saturation of phosphorylation and binding [113,239]. These numbers, summarized in Figure 8, are in excellent agreement with those obtained by quantification of fluorescence intensity at kinetochores [288]. In *S. cerevisiae*, early fluorescence measurements suggested ~8 KMN per centromeric CENP-A^Cse4^ nucleosome (or, more precisely, 8-fold higher fluorescence intensity for KMN subunits relative to CENP-A^Cse4^ in the cluster of 16 centromeres). As deletion of the CENP-T ortholog Cnn1 does not reduce kinetochore-localized Ndc80, which is in contrast to what is observed in human cells, how this stoichiometry is achieved remains at present unclear.

### 5.3. Temporal Framework of Kinetochore Assembly and Disassembly

The majority of CCAN subunits display continued centromere localization (defined as co-localization with CENP-A foci) during the cell cycle and, in most cases, negligible turnover rates [292,293,294,295]. Nonetheless, studies on the reciprocal dependencies of CCAN subunits during the cell cycle indicate clear differences between interphase and mitosis. For instance, CENP-C localization to centromeres appears to depend on CENP-HIKM subunits during interphase but not in mitosis (for instance see references [80,110,111,296]). New kinetochore incorporation of CENP-TW, CENP-N, and CENP-U may occur during DNA replication [177,297,298,299]. The kinetochore levels of CENP-OPQRU subunits, on the other hand, appear to decrease as cells enter mitosis [294,300]. The molecular basis for cell cycle-dependent regulation of CCAN subunit loading and stability is largely unknown. Phosphorylation likely plays a role in these processes [301]. 

Mitotic maturation of kinetochores focuses mainly on the creation of the outer kinetochore. In vertebrate cells, the KMN subunits are not localized with CENP-A foci in G1, but begin to be recruited in S-phase and G2, with the Ndc80 complex being the last to be recruited, due to its exclusion from the nuclear compartment and to its dependence on CDK activity for kinetochore localization [239,242,279,281,292,302]. In *D. melanogaster*, KMN assembly may only occur later, in prophase, but follows a similar assembly order, with the Mis12 complex and Knl1^Spc105^ assembly leading to Ndc80 complex recruitment after nuclear envelope breakdown [303]. As already clarified above, stabilization of the interaction of Mis12 with CENP-C might be an initiating trigger in KMN assembly on kinetochores. The components of the KMN network, on the other hand, disassemble from kinetochores at anaphase [292].

Probably the most dramatic physical transformation of regional kinetochores in metazoans is the formation of crescent-like shapes on their surface [7,8,24,25,26]. This phenomenon precedes end-on microtubule binding by the Ndc80 complex, and is believed to increase the likelihood of microtubule capture as well as to promote SAC signaling [8,27]. Proteins involved in this expansion had been previously localized to the kinetochore corona and include the microtubule motor CENP-E [26], the large (~400 kD) microtubule-binding protein CENP-F [8,304], and the dynein/dynactin motor complex along with its kinetochore targeting adaptors, the Rod-Zwilch-ZW10 (RZZ) complex [305]. RZZ’s largest subunit, Rod, is structurally related to clathrin [306], pointing to its oligomerization as a possible driver of corona expansion.

As clarified in more detail in the chapter by Maiato and colleagues [255], the RZZ complex is required for kinetochore recruitment of the minus-end directed motor cytoplasmic Dynein. This function of RZZ requires an additional protein named Spindly, which additionally acts as an adaptor capable of stimulating Dynein motility [232,307,308,309,310,311,312,313]. Kinetochore localization of Spindly requires the RZZ complex and farnesylation on a Cys residue near the C-terminus of Spindly [314,315]. Interestingly, the motor protein CENP-E and the microtubule-binding component CENP-F that also localize to the corona region of the kinetochore are both farnesylated [316,317].

Upon conversion of kinetochore attachments from lateral to end-on, i.e., when Ndc80 gains the upper hand in the attachment mechanism, the shape of kinetochores converts from an extended crescent to a smaller, plate-like appearance [8,27]. This shape change is associated with the motor-dependent release of the Dynein:Dynactin:Spindly:RZZ complex from kinetochores towards spindle poles [318,319,320,321,322,323,324,325]. The central spindle assembly checkpoint component, the Mad1:Mad2 complex, is also removed from kinetochores through this mechanism, effectively terminating SAC signaling by the kinetochore [325,326,327,328,329,330,331].

This brief section highlights that kinetochore assembly is regulated in response to cell cycle cues and kinetochore composition changes in response to microtubule attachment. Defining precisely how regulation operates in these two contexts is an important challenge for the future.

## 6. Organization of the Chromatin Foundation of the Kinetochore in Regional Centromeres

The above sections have focused on discrete, high-affinity stoichiometric physical interactions of kinetochore subunits that build the kinetochore on its chromatin foundation. This type of approach is helping define the assembly unit of the human kinetochore, which we propose has a CENP-A and H3.3 dinucleosome as its foundation (Figure 8). The remarkable progress made on understanding high-affinity stoichiometric interactions masks a relative paucity of information on how the regional or holocentric centromeres of most metazoan species, where a small number of CENP-A nucleosomes are interspersed with a large excess of H3 nucleosomes, are organized to form a multi-microtubule binding kinetochore. While it is generally assumed that individual CENP-A nucleosomes will recruit machinery resembling the unit kinetochore module described above [332,333], how these modules are clustered and organized to build a ‘surface’ with a high density of kinetochore components on a CENP-A nucleosomal platform is not known. In *S. pombe*, ~10–15 CENP-A^Cnp1^ nucleosomes in the 10 kb centromeric central core region drive the assembly of kinetochores capable of binding three microtubules [334,335]. At human centromeres, which bind ~20 microtubules, current estimates indicate that ~100 CENP-A nucleosomes are present but are dispersed in a large centromeric DNA region in which CENP-A is largely substoichiometric relative to H3 (roughly 1 to 25) [169]. Within such large repetitive centromere regions, CENP-A may be enriched in arrays forming discrete sub-domains [336,337,338]. How centromeric chromatin folds to expose the CENP-A domains for kinetochore assembly is currently unclear, and several models have been discussed [6]. Of note, studies in *S. cerevisiae* have suggested that the high density of cohesin flanking centromeres may aid extrusion of loops on the termini of which CENP-A^Cse4^ nucleosomes are present. This line of thinking, stimulated by models of chromatin polymer properties, suggests that the 16 kinetochores of budding yeast may be organized in a manner that resembles multi-microtubule binding kinetochores of metazoans [339,340,341] (Figure 7). In the holocentric nematode *Caenorhabditis elegans*, CENP-A occupies non-repetitive regions of 10–12 kb dispersed across about half of the genome and is excluded from loci that are transcribed in the germline and early embryo [342]; for a different view that suggests the presence of focused spots of functional CENP-A in *C. elegans*, in addition to the pattern summarize above, see [343]). Notably, in this holocentric species, CENP-A removal, but not CENP-C removal, causes chromosome structure/condensation defects [344], suggesting that CENP-A chromatin, independently of its role in kinetochore assembly, has a propensity to coordinate structural organization of chromatin, potentially via its intrinsic properties or via effectors other than those required to build a kinetochore.

As discussed in Section 5, the assembly unit of a kinetochore likely contains multiple Ndc80 complexes. Modulation by Aurora B phosphorylation of the binding affinity of each of these complexes for microtubules (see Section 4) likely gives rise to a considerable dynamic range of microtubule binding affinities. While kinetochores built on point centromeres bind a single microtubule, those built on regional centromeres bind multiple microtubules, but it is currently unclear whether a kinetochore module within regional kinetochores is associated with a single or multiple microtubules. In modeling studies, a random distribution of Ndc80 complexes on the kinetochore surface proves more versatile in comparison to the clustering into discrete regions binding a single microtubule [229,233]. Whether the scattering model is compatible with the kinetochore construction principles we have illustrated, however, remains to be clarified. 

This section illustrates a major gap in our understanding of kinetochore structure in a majority of species—how the dispersed and rare CENP-A nucleosomes at repetitive centromeres are collected and organized to form a base for kinetochore assembly. The properties of this complex chromatin domain are also relatively poorly understood. This gap in turn leaves open the important question how microtubule-binding sites are organized to efficiently capture and couple dynamic microtubules and how multiple microtubule-binding sites are coordinated. Recent work is beginning to reveal the importance of transcriptional activity at centromeres that may contribute to centromere structural organization. Repeats at centromeres are transcribed and active RNA polymerase II is detected at centromeres [345,346,347,348]. In addition, complexes implicated in transcription, most notably the FACT complex that remodels chromatin during transcription, has been co-purified with CENP-A chromatin in multiple independent studies, has been implicated in CENP-A loading, and was recently reported to interact with CENP-T [97,99,112,349]. Numerous concepts are currently being explored on the role of transcriptional activity, including creation of paused transcription sites, generation of centromeric RNAs that somehow act in centromere assembly, and generation of a chromatin state that is conducive to CENP-A loading [350,351,352].

## 7. The Propagation of Centromeric Chromatin

A fundamental requirement for the epigenetic specification of centromeres is that the pool of CENP-A be maintained through cell division. In the absence of specific recognition of the underlying DNA sequence, this process is likely to be directed by the existing pool of CENP-A [72]. In vertebrates, incorporation of new CENP-A at centromeres occurs after mitotic exit, from mitotic telophase till early G1 phase [135,153,177,299,353]. The CENP-A pool is then distributed, without new incorporation, to the sister chromatids during DNA replication, with the resulting gaps being probably filled with histone H3.3 [354]. Thus, sister chromatids enter mitosis with half as much CENP-A as that present on the parental chromosome before replication. In the absence of sequence-specific interactions with DNA, a crucial unresolved question is if CENP-A positional information is retained during DNA replication, when the nucleosome structure of the centromere is likely to become temporarily perturbed, and if so, how. Once incorporated into chromatin, CENP-A is not further evicted, showing negligible dissociation kinetics [141,152,153,295,355].

**Figure 9:**
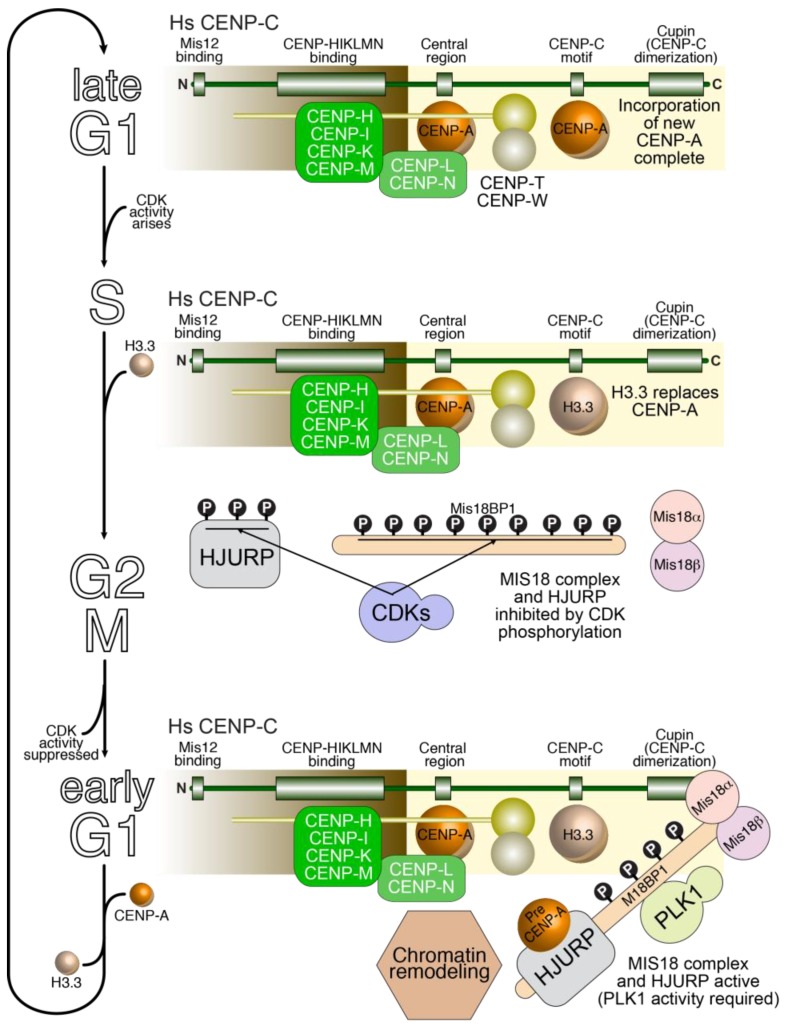
Cell cycle-regulated replenishment of CENP-A nucleosomes New. CENP-A incorporation takes place after mitotic exit, early in G1 phase (*bottom*). It is driven by an existing, active CENP-A nucleosome (e.g., because bound to CCAN subunits), and directed on a neighboring nucleosome. Here, we hypothesize that the neighboring nucleosome in humans is already bound to the CENP-C motif of CENP-C. An active MIS18 complex, including M18BP1, recruits HJURP, which binds to pre-nucleosomal CENP-A. The C-terminal region of CENP-C has been implicated in this reaction, which also requires PLK1 activity. A chromatin-remodeling factor and other chromatin-associated factors promote extraction of H3.3 and its replacement with CENP-A through an ATP-dependent reaction. The resulting configuration (*top*) persists until DNA replication (S-phase), when CENP-A “vacancies” caused by distribution of CENP-A to the sister chromatids during DNA replication, are filled with H3.3. This configuration then persists through the rest of the cell cycle until mitotic exit, because the CENP-A loading machinery is inhibited by CDK activity (*middle*).

Several factors involved in the deposition of new CENP-A have emerged [4,6] (Figure 9). The Mis18 complex, first identified based on a mutant in *S. pombe*, includes Mis18 (orthologous to human paralogs Mis18α and Mis18β) and Mis16 (orthologous to human proteins RbAp46/48, and acting as histone chaperones in several histone modification complexes) [339,356,357,358,359]. Mis18 forms oligomers in *S. pombe* and humans [360,361]. The Mis18 complex further interacts with M18BP1 (Mis18 binding protein 1, also known as Knl2) [356,357,358,362,363]. No Mis18BP1 has been identified in *S. pombe*, but two recently identified proteins, Mis19/Eic1 and Mis20/Eic2 may act as functional orthologs of M18BP1 in this organism [364,365].

The Mis18 complex, in conjunction with M18BP1, mediates a cell-cycle-regulated interaction with kinetochores that ultimately promotes the localization of a CENP-A selective histone chaperone known in vertebrates as HJURP (Holliday Junction-Recognizing Protein) [366,367,368]. Functional orthologs with limited sequence similarity to HJURP exist in *S. cerevisiae*, *S. pombe*, and *D. melanogaster* (named Scm3 and Cal1) [369,370,371,372,373,374,375,376,377,378,379]. HJURP, which may form functional dimers [380], contains an N-terminal Scm3-homology domain that binds pre-nucleosomal CENP-A [73,75,158,381,382,383,384]. Its central region, on the other hand, mediates binding to M18BP1 and is required for kinetochore recruitment [73,385,386]. The precise mechanism of kinetochore recruitment of the Mis18 complex and HJURP remains unclear, but interactions with CCAN subunits, including CENP-I^Mis6^ and CENP-C, have been identified [75,158,374,378,385,387,388,389,390,391]. In *S. cerevisiae*, on the other hand, Scm3 is recruited through an interaction with the CBF3 complex [371].

CENP-A deposition is tightly coordinated with cell cycle progression in a manner distinct from canonical histone H3, which is deposited concomitantly with DNA replication in S-phase. In vertebrates, incorporation of new CENP-A is limited to telophase and early G1, when CDK activity is suppressed [392,393,394,395]. The increase in CDK activity that precedes the initiation of DNA replication may be sufficient for inhibition of CENP-A deposition, implicating pre-mitotic Cdk2 kinase activity in complex with Cyclin E and Cyclin A as potential negative regulators of this process. A major target of this regulation is M18BP1, which CDKs phosphorylate at multiple sites, preventing its interaction with Mis18 subunits and its kinetochore recruitment [392,395,396]. Inhibition of CENP-A deposition through phosphorylation of HJURP and CENP-A has also been described [393,394,396,397]. 

The activity of another kinase, Polo-like Kinase 1 (Plk1), on the other hand, is required for the incorporation of new CENP-A [395]. Suppression of CDK activity in the G2 phase of the cell cycle results in ectopic and Plk1-dependent incorporation of CENP-A, suggesting that Plk1 is required for CENP-A deposition but does not contribute to cell cycle phase coordination. Like CDKs, Plk1 also targets the Mis18:M18BP1 complex, but the precise interactions controlled by this kinase remain to be discovered.

The molecular mechanism of new CENP-A deposition remains unclear [5]. Factors implicated in CENP-A deposition, or in its stable maintenance at centromeres, include the chromatin remodeling FACT complex, the histone chaperone NPM1/nucleophosmin, the GTPase-activating protein MgcRacGAP, And-1, and Condensin II [99,112,349,368,398,399,400,401,402,403,404,405]. The RSF and MgcRacGAP, the latter identified as a binding partner of [363] P1/Knl2, have been implicated specifically in the maintenance of newly incorporated CENP-A [400]. Several DNA and histone post-translational modifications have been implicated in CENP-A loading, including DNA methylation by DNMT3B, possibly through interactions with the C-terminal region of CENP-C and with Mis18α [406,407], acetylation [402,408,409], and histone H3K4 dimethylation [410,411].

As clarified above, during DNA replication the chromosome levels of CENP-A are reduced by 50%. It has been proposed that the CENP-A ‘vacancies’ generated at this stage are filled by placeholder histone H3.3 [354]. New CENP-A deposition in telophase and early G1 may therefore require the eviction of the H3.3 previously used to fill the CENP-A vacancy, and its replacement with CENP-A. Existing kinetochores provide a recruitment platform for the CENP-A deposition machinery, limiting its function to existing centromeres. The amount of CENP-A on each chromosome appears to be constant through generations, implying that each CENP-A may only trigger a single cycle of CENP-A incorporation [412,413]. Like other histone replacement reactions [414], rapid replacement of H3 with CENP-A is likely to require a source of energy. The ATPase carrying out the replacement has not yet been identified with certainty. Two chromatin-remodeling enzymes, Chd1 and RSF, have been implicated in CENP-A deposition in human cells [99,112,401], while Chd1 appears to be dispensable for CENP-A deposition in *D. melanogaster* [415].

If the CENP-A deposition reaction implies the substitution of H3.3 with CENP-A, a crucial question is how the CENP-A deposition machinery targets a specific H3.3 nucleosome neighboring CENP-A. Likely, there are specific molecular features that designate it as the nucleosome to be replaced. We suggest that the C-terminal region of CENP-C is responsible for this labeling function (Figure 9). As explained in Section 3, the N-terminal region of CENP-C, comprising a succession of binding sites for Mis12, the CENP-CHIKMLN complex, and CENP-A, is required for kinetochore assembly [110,165]. However, CENP-C contains a second nucleosome-binding motif, the CENP-C motif, and we surmise that it mediates the association with a second nucleosome. Our speculative working model is that the structure recognized by the CENP-A deposition machinery is a CENP-A:H3 dinucleosome in which CENP-C acts as a bridge between the two nucleosomes. After eviction of H3 and deposition of CENP-A, the deposition reaction is complete and the deposition machinery dissociates.

This model predicts that the core kinetochore module in regional kinetochores is a dinucleosome structure. One of the nucleosomes has permanent identity as CENP-A, being bound to the N-terminal region of CENP-C where all CCAN subunits stably assemble. The second one, bound to the C-terminal region of CENP-C (implicated in recruitment of the CENP-A deposition machinery, as observed above), toggles between two identities (H3 and CENP-A) during the cell cycle (Figure 9). While this hypothesis requires considerable further scrutiny, it is consistent with studies showing that human CENP-A nucleosomes are enriched on units of ~340 bps consisting of two α-satellite repeats separated by a CENP-B box, and on which CENP-B, CENP-C, and CENP-T also appear to co-exist with two CENP-A molecules in a single complex [77,78,416]. Dimeric repeat units are observed in other mammals [417]. In *S. cerevisiae*, where a specific DNA motif defines the centromere, only the organization of the “permanent” CENP-A nucleosome is preserved, while CBF3 functionally replaces the second nucleosome by providing direct DNA recognition and by recruiting the Scm3 chaperone. In this organism, deposition of new Cse4 occurs concomitantly with DNA replication [355,418].

## 8. Unconventional Kinetochores

Our discussion above has focused on kinetochore assembly that occurs on CENP-A nucleosomal chromatin. The picture we present, in which CENP-A nucleosomal chromatin recruits CCAN components that recruit KMN complexes that in turn recruit other microtubule-interacting proteins and SAC machinery, is true in budding and fission yeast, *D. melanogaster*, *C. elegans*, and vertebrates. However, notable exceptions to this picture have been found and have the potential to reveal core principles on how kinetochores are built to couple to microtubules and ensure chromosome segregation. The most striking exception found to date is in kinetoplastids, where proteins that are homologous to the majority of the kinetochore proteins we discussed here (CENP-A, CCAN, KMN) have not been found. Based on tagging and proteomic analysis, 20 kinetochore proteins have been identified in the kinetoplastid *Trypanosoma brucei* [419] and their detailed characterization should reveal how this divergent kinetochore is built and operates. Based on weak homology to the Ndc80 and Nuf2 coiled-coils, a very recent study identified a new kinetochore-localized protein in *T. brucei* that is important for chromosome segregation [420]. Although this protein lacks the calponin homology (CH) domains of Ndc80 and Nuf2 that mediate microtubule binding, the sequence homology in the coiled coil suggests divergence from an ancestral Ndc80/Nuf2-like protein. Kinetoplastids are classified as belonging to a diverse group of eukaryotes (known as the Excavata) that are distinct from the group that contains the commonly studied model organisms (known as the Opisthokonta). *Giardia intestinalis*, a diplomonad that is also classified as belonging to the Excavata group, does have a CENP-A-like histone and KMN proteins. Thus, kinetoplastids exhibit extreme divergence of ancestral components together with emergence of new kinetochore machinery, the reasons for which are currently mysterious. A second exception to the canonical kinetochore assembly pathway is observed in holocentric insect species, which have independently arisen multiple times. In at least four independent lineages of insects where holocentricity evolved, CENP-A and CENP-C appear to have been lost but KMN components are still present [62]. Thus, these holocentric insect lineages appear to build their kinetochores on a different foundation than CENP-A. This finding echoes earlier work in *C. elegans*, where the mitotic kinetochore requires CENP-A for assembly and follows the canonical pathway but the meiotic kinetochore in oocytes is built independently of CENP-A and CENP-C [421]. Defining how the holocentric insect kinetochore is built independently of CENP-A and assessing whether an epigenetic inheritance mechanism, similar to what is observed with a CENP-A based kinetochore foundation, is also operating in these species will be very revealing.

## 9. Conclusions

In this review, we have focused on the structural organization of the kinetochore. It is with this largely evolutionarily conserved scaffold that the kinetochore performs its complex functions, including error correction, spindle assembly checkpoint control, and chromosome alignment and segregation. While a detailed mechanistic understanding of kinetochore dynamics is still lacking, our grasp of kinetochore architecture inspires molecular hypotheses on the molecular changes to kinetochore architecture that distinguish microtubule-bound and -unbound states and their signaling properties.
